# Supplementary material for: Acridine Based N-Acylhydrazone Derivatives as Potential Anticancer Agents: Synthesis, Characterization and ctDNA/HSA Spectroscopic Binding Properties
Source: Molecules. 2022 Apr 30;27(9):2883. doi: 10.3390/molecules27092883 (PMC9100673; doi:10.3390/molecules27092883)
Supplement: Supplementary file 1 [file molecules-27-02883-s001.zip › molecules-1673302-supplementary.pdf]

SUPPLEMENTARY INFORMATION

Acridine Based *N*-Acylhydrazone Derivatives as Potential Anticancer Agents: Synthesis, Characterization and ctDNA/HSA Spectroscopic Binding Properties

Mária Vilková [1], Monika Hudáčová [2], Nikola Palušeková [2], Rastislav Jendželovský [3], Miroslav Almáši [4], Tibor Béres [5], Peter Fedoročko [3] and Mária Kožurková [2,*]

[1] NMR Laboratory, Institute of Chemical Sciences, Faculty of Science, P. J. Šafárik University in Košice, Moyzesova 11, 041 67 Košice, Slovakia; maria.vilkova@upjs.sk
[2] Department of Biochemistry, Institute of Chemical Sciences, Faculty of Science, P. J. Šafárik University in Košice in Košice, Moyzesova 11, 041 67 Košice, Slovakia; monika.hudacova@student.upjs.sk (M.H.); nikola.palusekova@upjs.sk (N.P.);
[3] Department of Cellular Biology, Institute of Biology, Faculty of Science, P. J. Šafárik University in Košice, Moyzesova 11, 041 67 Košice, Slovakia; peter.fedorocko@upjs.sk (P.F.), rastislav.jendzelovsky@upjs.sk (R.J.)
[4] Department of Inorganic Chemistry, Institute of Chemical Sciences, Faculty of Science, P. J. Šafárik University in Košice, Moyzesova 11, 041 67 Košice, Slovakia; miroslav.almasi@upjs.sk
[5] Czech Advanced Technology and Research Institute, Centre of the Region Hana for Biotechnological and Agricultural Research, Palacký University, Šlechtitelů 241/27, 779 00 Olomouc, Czech Republic; tibor.beres@upol.cz

Contents

1 GENERAL INFORMATION 3

2 NMR SPECTRA OF DERIVATIVES 3A–E AND 7B–D. 7
1.1 *N*'-[(*E*)-Acridin-4-yl)methylidene]benzohydrazide (3a) 7
1.2 *N*'-[(*E*)-Acridin-4-yl)methylidene]-4-fluorobenzohydrazide (3b) 9
1.3 *N*'-[(*E*)-Acridin-4-yl)methylidene]-4-chlorobenzohydrazide (3c) 11
1.4 *N*'-[(*E*)-Acridin-4-yl)methylidene]-4-bromobenzohydrazide (3d) 13
1.5 *N*'-[(*E*)-Acridin-4-yl)methylidene]-4-methoxybenzohydrazide (3e) 15
1.6 *N*'-[(*E*)-4-fluorophenylmethylidene]acridine-4-carbohydrazide 7b 17
1.7 *N*'-[(*E*)-4-chlorophenylmethylidene]acridine-4-carbohydrazide 7c 19
1.8 *N*'-[(*E*)-4-bromophenylmethylidene]acridine-4-carbohydrazide 7d 21

3 HR MS SPECTRA OF DERIVATIVES 3A–E AND 7B–D 23
1.9 *N*'-[(*E*)-Acridin-4-yl)methylidene]benzohydrazide (3a) 23
1.10 *N*'-[(*E*)-Acridin-4-yl)methylidene]-4-fluorobenzohydrazide (3b) 24
1.11 *N*'-[(*E*)-Acridin-4-yl)methylidene]-4-chlorobenzohydrazide (3c) 25
1.12 *N*'-[(*E*)-Acridin-4-yl)methylidene]-4-bromobenzohydrazide (3d) 26
1.13 *N*'-[(*E*)-Acridin-4-yl)methylidene]-4-methoxybenzohydrazide (3e) 27
1.14 *N*'-[(*E*)-4-Fluorophenylmethylidene]acridine-4-carbohydrazide (7b) 28
1.15 *N*'-[(*E*)-4-Chlorophenylmethylidene]acridine-4-carbohydrazide (7c) 29
1.16 *N*'-[(*E*)-4-Bromophenylmethylidene]acridine-4-carbohydrazide (7d) 30

4 IR SPECTRA OF COMPOUNDS 3A–D AND 7B, 7D 31
1.17 *N*'-[(*E*)-Acridin-4-yl)methylidene]benzohydrazide (3a) 31
1.18 *N*'-[(*E*)-Acridin-4-yl)methylidene]-4-fluorobenzohydrazide (3b) 32
1.19 *N*'-[(*E*)-Acridin-4-yl)methylidene]-4-chlorobenzohydrazide (3c) 32
1.20 *N*'-[(*E*)-Acridin-4-yl)methylidene]-4-bromobenzohydrazide (3d) 32

1.21 *N'*-[(*E*)-4-Fluorophenylmethylidene]acridine-4-carbohydrazide (7b) 33
1.22 *N'*-[(*E*)-4-Bromophenylmethylidene]acridine-4-carbohydrazide (7d).................................. 33

5 HAS BINDING EXPERIMENTS 34

1 General information

Table S1. Physico-chemical data for compounds **3a–e** and **7b–d**.

	Name	Melting point [°C]	Color	Formula	Mass (calcd./found)	Yield [mg]	Yield [%]
**3a**	*N'*-[(*E*)-(Acridin-4-yl)methylidene]benzohydrazide	235–236	bright yellow	$C_{21}H_{16}N_3O$	326.1293/326.1291	141	90
**3b**	*N'*-[(*E*)-(Acridin-4-yl)methylidene]-4-fluorobenzohydrazide	229–230	bright yellow	$C_{21}H_{15}N_3OF$	344.1199/326.1201	135	82
**3c**	*N'*-[(*E*)-(Acridin-4-yl)methylidene]-4-chlorobenzohydrazide	266–267	bright yellow	$C_{21}H_{15}N_3OCl$	360.0904/360.0905	158	91
**3d**	*N'*-[(*E*)-(Acridin-4-yl)methylidene]-4-bromobenzohydrazide	269–270	bright yellow	$C_{21}H_{15}N_3OBr$	404.0399/404.0396	143	73
**3e**	*N'*-[(*E*)-(Acridin-4-yl)methylidene]-4-methoxybenzohydrazide	249–250	bright yellow	$C_{22}H_{18}N_3O_2$	356.1399/356.1398	172	82
**7b**	*N'*-[(*E*)-(4-Fluorophenyl)methylidene]acridine-4-carbohydrazide	255–256	bright yellow	$C_{21}H_{15}N_3OF$	344.1199/344.1196	130	90
**7c**	*N'*-[(*E*)-(4-Chlorophenyl)methylidene]acridine-4-carbohydrazide	243–244	bright yellow	$C_{21}H_{15}N_3OCl$	360.0904/360.0901	121	80
**7d**	*N'*-[(*E*)-(4-Bromophenyl)methylidene]acridine-4-carbohydrazide	240–241	bright yellow	$C_{21}H_{15}N_3OBr$	404.0399/404.0395	140	82

Table S2. ^{1}H NMR (DMSO-d_6) data for compounds $E_{C=N}Z_{C(O)-N}$-**3a–e**. Chemical shifts are reported in δ (ppm) relative to DMSO-d_5 signal (2.50 ppm), multiplicities, and coupling constants (Hz) are given in parentheses.

	**3a**	**3b**	**3c**	**3d**	**3e**
**H-2**	12.25 (s)	12.26 (s)	12.30 (s)	12.30 (s)	12.12 (s)
**H-4**	9.93 (s)	9.91 (s)	9.92 (s)	9.92 (s)	9.90 (s)
**H-1'**	8.30 (d, 8.4)	8.31 (d, 8.6)	8.31 (d, 7.6)	8.30 (d, 9.2)	8.29 (d, 8.3)
**H-2'**	7.76 (t, 7.2)	7.76 (t, 7.7)	7.76 (t, 7.7)	7.75 (t, 7.2)	7.75 (t, 7.7)
**H-3'**	8.51 (d, 7.3)	8.51 (d, 6.7)	8.51 (d, 5.9)	8.50 (d, 6.3)	8.49 (d, 6.5)
**H-5'**	8.19 (d, 8.7)	8.19 (d, 8.5)	8.19 (d, 9.3)	8.19 (d, 8.8)	8.20 (d, 8.7)
**H-6'**	7.93 (ddd, 8.8, 6.5, 1.4)	7.93 (ddd, 8.5, 6.6, 1.4)	7.93 (ddd, 8.5, 6.5, 1.4)	7.93 (ddd, 8.5, 6.6, 1.4)	7.93 (ddd, 8.4, 6.5, 1.4)
**H-7'**	7.68 (ddd, *J* 8.0, 6.6, 1.1)	7.68 (ddd, 8.1, 6.5, 1.0)	7.68 (ddd, 8.1, 6.6, 1.1)	7.67 (t, 8.0)	7.68 (t, 7.5)
**H-8'**	8.23 (d, 8.3)	8.23 (d, 8.7)	8.23 (d, 8.6)	8.23 (d, 8.5)	8.23 (d, 8.3)
**H-9'**	9.22 (s)	9.23 (s)	9.23 (s)	9.22 (s)	9.22 (s)
**H-2'',6''**	8.04 (d, 7.1)	8.11 (d, 8.4)	8.06 (d, 8.5)	7.99 (d, 8.5)	8.03 (d, 8.4)
**H-3'',5''**	7.56 (t, 7.5)	7.40 (d, 8.7)	7.64 (d, 8.5)	7.78 (d, 8.5)	7.09 (d, 8.8)
**H-4''**	7.63 (t, 7.3)	-	-	-	
**CH_3**	-	-	-	-	3.86 (s)

Table S3. ^{13}C NMR (DMSO-d_6) data for compounds $E_{C=N}Z_{C(O)-N}$-**3a–e**. Chemical shifts are reported in δ (ppm) relative to DMSO-d_6 signal (39.52 ppm).

	**3a**	**3b**	**3c**	**3d**	**3e**
**C-1**	163.1	162.1	162.1	162.2	162.5
**C-4**	144.7	144.8	145.0	145.1	144.0
**C-1'**	130.5	130.6	130.6	130.6	130.4
**C-2'**	125.7	125.7	125.7	125.7	125.7
**C-3'**	126.8	126.8	126.8	126.8	126.6
**C-4'**	131.0	130.9	130.9	130.9	131.1
**C-5'**	128.8	128.8	128.8	128.8	128.8
**C-6'**	131.2	131.3	131.3	131.3	131.2
**C-7'**	126.2	126.2	126.2	126.2	126.2
**C-8'**	128.7	128.7	128.7	128.7	128.7
**C-9'**	137.1	137.1	137.1	137.1	137.1
**C-8'a**	126.2	126.2	126.2	126.2	126.2
**C-9'a**	126.2	126.2	126.2	126.2	126.2
**C-4'a**	146.2	146.2	146.2	146.2	146.2
**C-10'a**	147.9	147.9	147.9	147.9	147.9
**C-1''**	133.4	129.8	132.1	132.4	125.4
**C-2'',6''**	127.8	130.5	129.7	129.9	129.7
**C-3'',5''**	128.4	128.6	128.6	131.5	113.7
**C-4''**	131.8	164.2	136.6	125.6	162.0
**CH_3**	-	-	-	-	55.4

Table S4. ^{15}N NMR (DMSO-d$_6$) data for compounds $E_{C=N}Z_{C(O)–N}$-**3a–e**. Chemical shifts are reported in δ (ppm) relative to external CH_3NO_2 (0.00 ppm).

	**3a**	**3b**	**3c**	**3d**	**3e**
**N-2**	-206.0	-206.3	-206.0	-206.0	-207.3
**N-3**	-57.3	-57.6	-57.9	-58.0	-56.6
**N-10'**	-83.3	nd	nd	nd	-83.3

Table S5. ^{1}H NMR (acetone-d$_6$) data for compounds $E_{C=N}Z_{C(O)–N}$-**7b–d**. Chemical shifts are reported in δ (ppm) relative to acetone-d$_5$ signal (2.50 ppm), multiplicities, and coupling constants (Hz) are given in parentheses.

	***Z*N–N-7b**	***E*N–N-7b**	***Z*N–N-7c**	***E*N–N-7c**	***Z*N–N-7d**	***E*N–N-7d**
**H-2**	14.98 (s)	14.98 (s)	15.00 (s)	15.00 (s)	15.05 (s)	15.05 (s)
**H-4**	8.88 (s)	8.86 (s)	8.85 (s)	8.83 (s)	8.85 (s)	8.84 (s)
**H-1'**	8.44 (dd, 8.3, 1.6)	8.44 (dd, 8.3, 1.6)	8.41 (dd, 8.3, 1.6)	8.41 (dd, 8.3, 1.6)	8.44 (dd, 8.3, 1.6)	8.44 (dd, 8.3, 1.6)
**H-2'**	7.82 (dd, 8.3, 7.1)	7.82 (dd, 8.3, 7.1)	7.80 (dd, 8.3, 7.1)	7.80 (dd, 8.3, 7.1)	7.82 (dd, 8.3, 7.1)	7.82 (dd, 8.3, 7.1)
**H-3'**	9.00 (d, 7.0)	9.00 (d, 7.0)	8.98 (d, 7.0)	8.98 (d, 7.0)	9.00 (d, 7.0)	9.00 (d, 7.0)
**H-5'**	8.49 (d, 8.8)	8.49 (d, 8.8)	8.46 (d, 8.8)	8.46 (d, 8.8)	8.49 (d, 8.5)	8.49 (d, 8.5)
**H-6'**	8.02 (m)	8.02 (m)	7.99 (ddd, 8.4, 6.6, 1.2)	7.99 (ddd, 8.4, 6.6, 1.2)	8.01 (ddd, 8.4, 6.6, 1.2)	8.01 (ddd, 8.4, 6.6, 1.2)
**H-7'**	7.75 (ddd, 8.0, 6.6, 1.0)	7.75 (ddd, 8.0, 6.6, 1.0)	7.73 (ddd, 8.4, 6.6, 1.2)	7.73 (ddd, 8.4, 6.6, 1.2)	7.74 (ddd, 8.4, 6.6, 1.2)	7.74 (ddd, 8.4, 6.6, 1.2)
**H-8'**	8.27 (d, 8.5)	8.27 (d, 8.5)	8.24 (d, 8.5)	8.24 (d, 8.5)	8.26 (d, 8.5)	8.26 (d, 8.5)
**H-9'**	9.32 (s)	9.32 (s)	9.28 (s)	9.28 (s)	9.32 (s)	9.32 (s)
**H-2'',6''**	7.99 (dd, 8.8, 5.6)	7.99 (dd, 8.8, 5.6)	7.94 (d, 8.5)	7.94 (d, 8.5)	7.88 (d, 8.5)	7.88 (d, 8.5)
**H-3'',5''**	7.27 (t, 8.7)	7.27 (t, 8.7)	7.52 (d, 8.5)	7.52 (d, 8.5)	7.69 (d, 8.5)	7.69 (d, 8.5)

Table S6. ^{13}C NMR (acetone-d$_6$) data for compounds $E_{C=N}Z_{C(O)–N}$-**7b–d**. Chemical shifts are reported in δ (ppm) relative to acetone-d$_6$ signal (39.52 ppm).

	***Z*N–N-7b**	***E*N–N-7b**	***Z*N–N-7c**	***E*N–N-7c**	***Z*N–N-7d**	***E*N–N-7d**
**C-1**	162.6	162.5	162.6	162.7	162.5	162.6
**C-4**	147.7	147.7	147.6	147.5	147.6	147.6
**C-1'**	134.4	134.4	134.5	134.4	134.5	134.5
**C-2'**	126.4	126.4	126.3	126.3	126.3	126.4
**C-3'**	136.8	136.8	136.8	136.8	136.9	136.9
**C-4'**	128.9	128.9	128.8	128.8	128.8	128.8
**C-5'**	129.6	129.6	129.6	129.6	129.6	129.6
**C-6'**	132.9	133.0	132.9	133.0	132.9	133.0
**C-7'**	127.6	127.6	127.6	127.6	127.6	127.6
**C-8'**	129.5	129.5	129.4	129.4	129.4	129.4
**C-9'**	140.0	140.0	140.0	140.0	140.0	140.0
**C-8'a**	127.2	127.2	127.2	127.2	127.2	127.2
**C-9'a**	128.0	128.0	127.9	127.9	127.9	127.9
**C-4'a**	146.7	146.7	146.6	146.6	146.6	146.6
**C-10'a**	148.5	148.5	148.5	148.4	148.5	148.4
**C-1''**	132.7	132.7	135.1	135.1	135.5	135.5
**C-2'',6''**	130.3	130.3	129.8	129.8	130.0	130.0
**C-3'',5''**	116.5	116.5	129.8	129.8	132.7	132.7
**C-4''**	164.7	164.7	136.0	136.0	124.3	124.3

Table S7. ^{15}N NMR (acetone-d_6) data for compounds $E_{C=N}Z_{C(O)–N}$-**7b–d**. Chemical shifts are reported in δ (ppm) relative to external CH_3NO_2 (0.00 ppm).

	$\boldsymbol{Z_{N–N}}$**-7b**	$\boldsymbol{E_{N–N}}$**-7b**	$\boldsymbol{Z_{N–N}}$**-7c**	$\boldsymbol{E_{N–N}}$**-7c**	$\boldsymbol{Z_{N–N}}$**-7d**	$\boldsymbol{E_{N–N}}$**-7d**
**N-2**	-200.6	-199.5	-200.0	-199.0	-199.6	-198.9
**N-3**	-60.2	-59.7	-57.9	-57.3	-57.5	-57.2
**N-10'**	-95.9	-95.9	-96.3	-96.3	-96.3	-96.3

Table S8. Selected heteronuclear spin-spin coupling constants for derivatives $E_{C=N}Z_{C(O)–N}$-**3a–e** (DMSO-d_6) and $E_{C=N}Z_{C(O)–N}$-**7b–d** (acetone-d_6).

J [Hz]	**3a**	**3b**	**3c**	**3d**	**3e**	**7b**	**7c**	**7d**
$^1\boldsymbol{J}_{\mathbf{C4H4}}$	168.0	168.0	168.0	168.6	167.4	162.6	162.6	-
$^2\boldsymbol{J}_{\mathbf{C1H2}}$	10.8	-	11.4	-	-	-	-	-
$^1\boldsymbol{J}_{\mathbf{N2H2}}$	93.0	93.6	93.0	94.8	93.6	91.8	91.8	91.8

2 NMR spectra of derivatives 3a–e and 7b–d.

1.1 *N*'-[(*E*)-Acridin-4-yl)methylidene]benzohydrazide (3a)

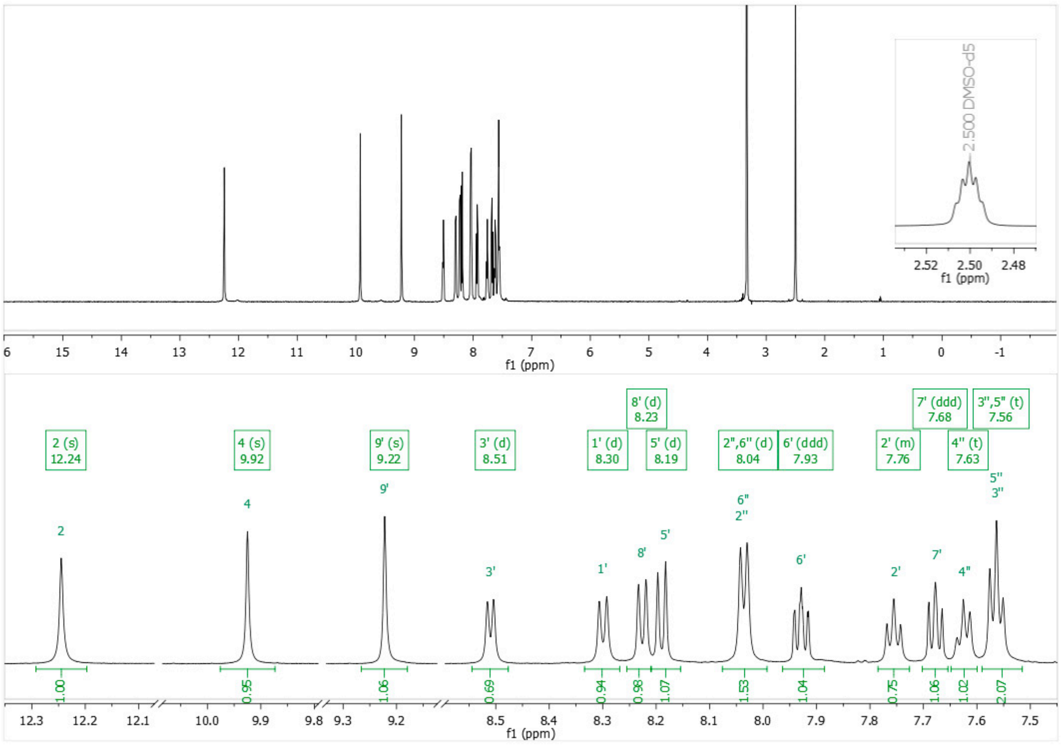

^{1}H NMR spectrum (600 MHz, DMSO-d$_6$) of the derivative **3a**.

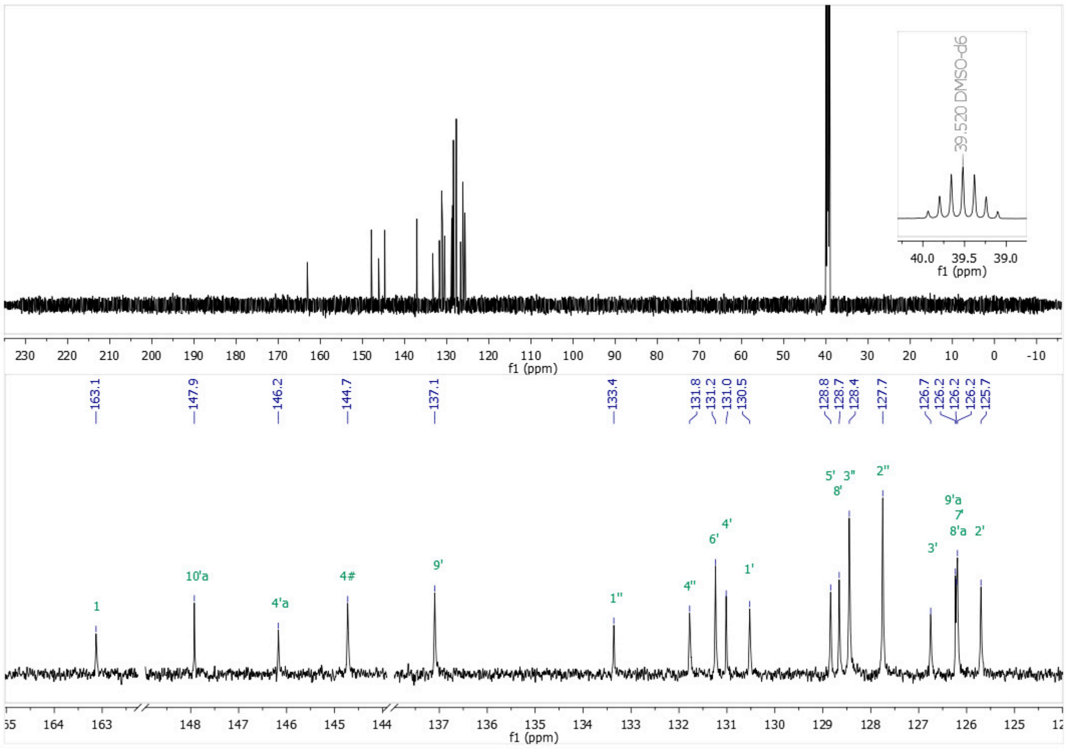

^{13}C NMR spectrum (151 MHz, DMSO-d$_6$) of the derivative **3a**.

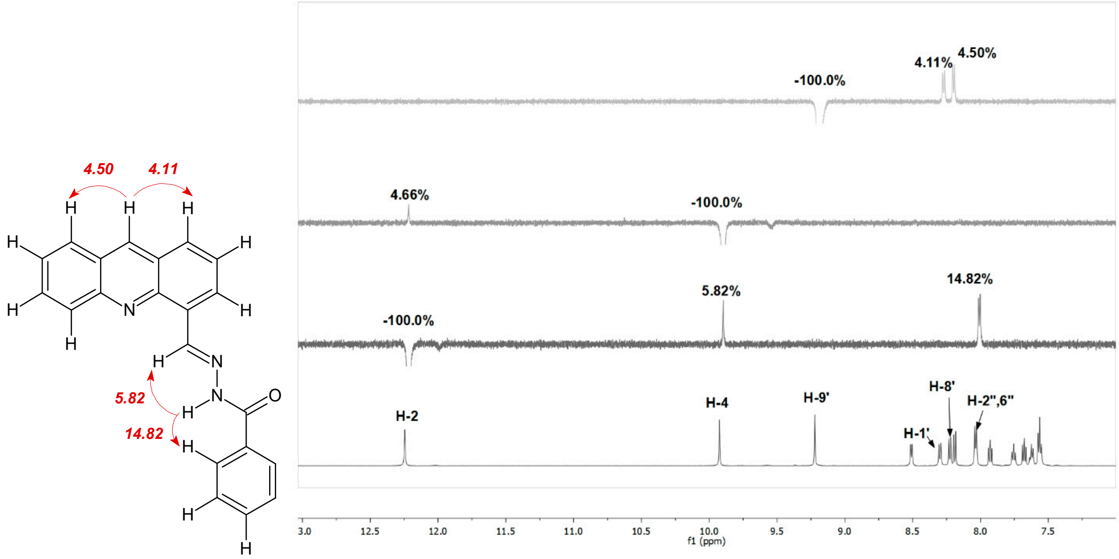

1D NOESY spectra of derivative $E_{C=N}Z_{C(O)-N}$-**3a**.

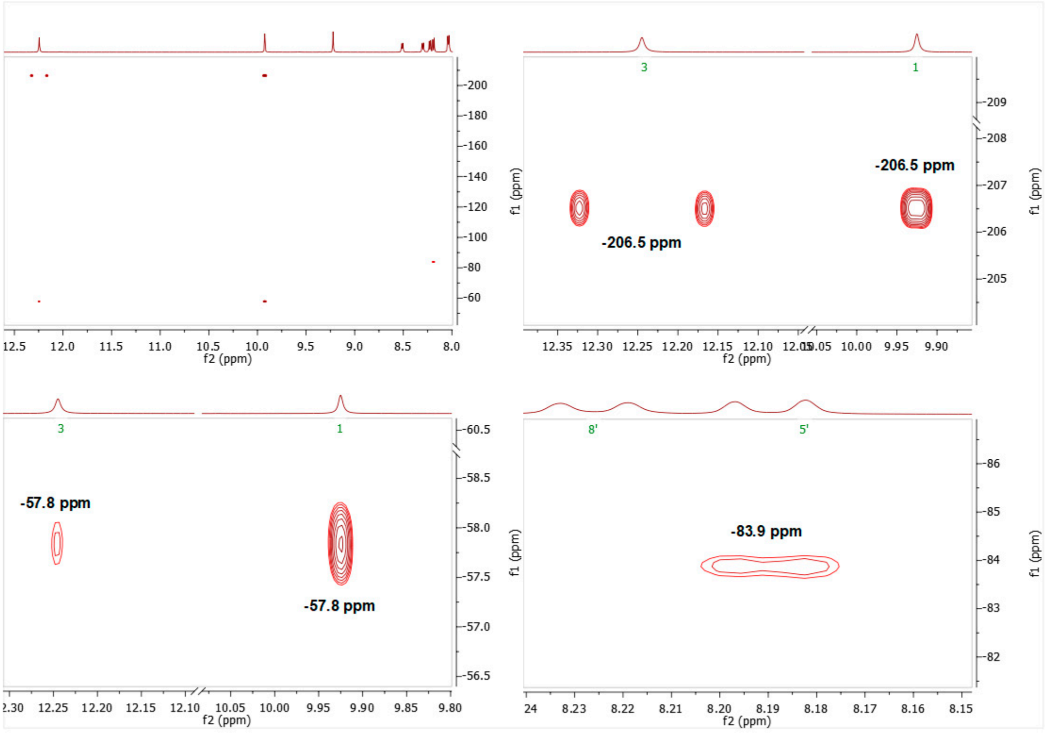

^{1}H,^{15}N-HMBC spectrum (600/61 MHz, DMSO-d$_6$) of the derivative **3a**.

1.2 *N'*-[(*E*)-Acridin-4-yl)methylidene]-4-fluorobenzohydrazide (3b)

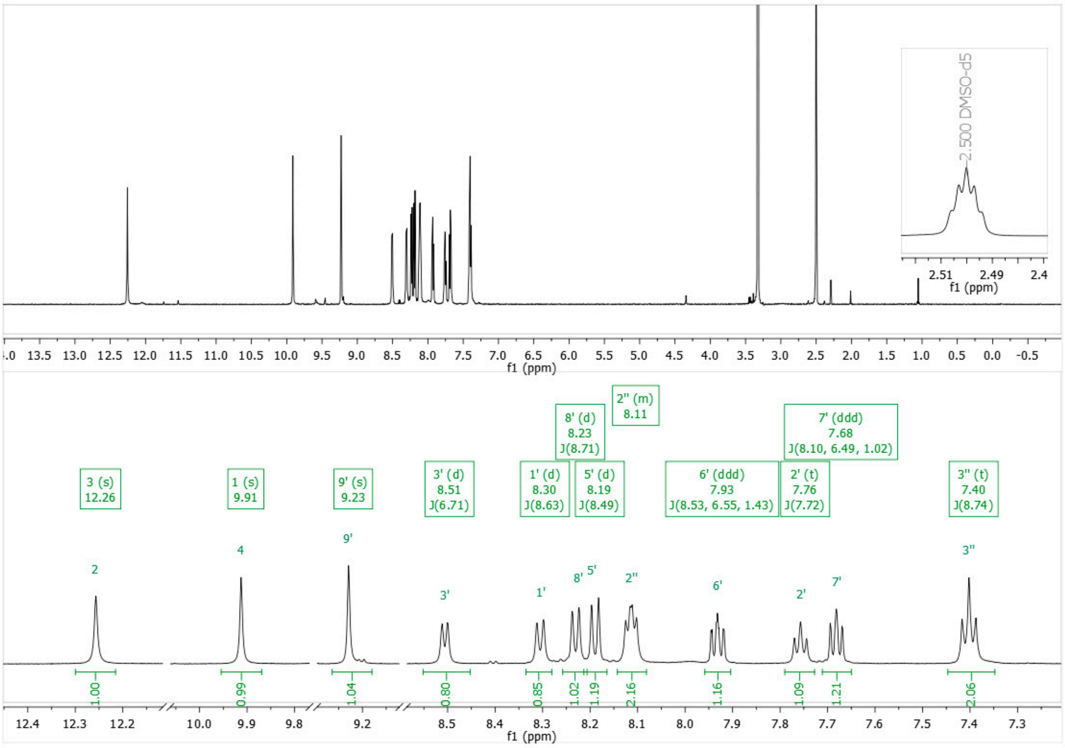

^{1}H NMR spectrum (600 MHz, DMSO-d$_6$) of the derivative **3b**.

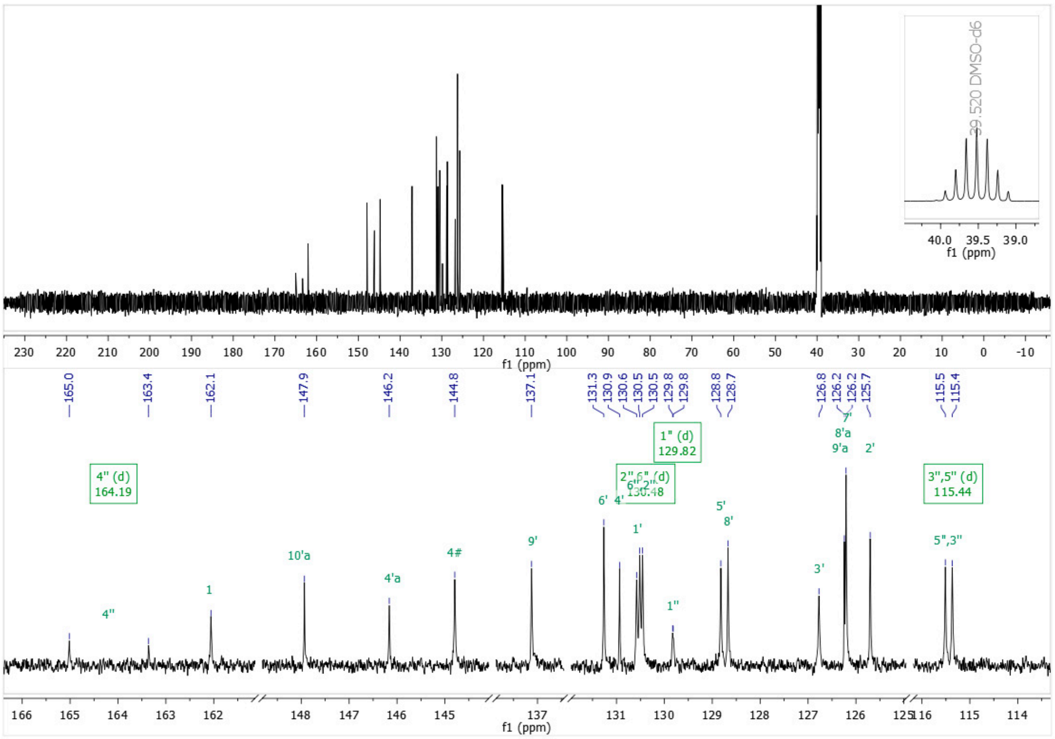

^{13}C NMR spectrum (151 MHz, DMSO-d$_6$) of the derivative **3b**.

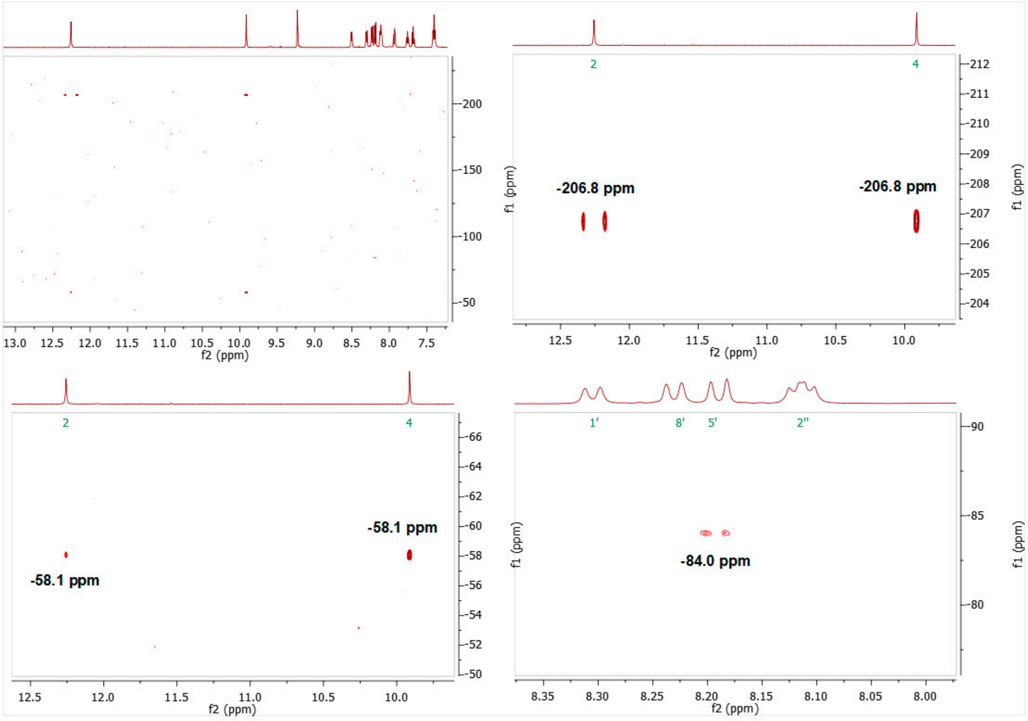

$^{1}H,^{15}N$-HMBC spectrum (600/61 MHz, DMSO-d_6) of the derivative **3b**.

1.3 *N'*-[(*E*)-Acridin-4-yl)methylidene]-4-chlorobenzohydrazide (3c)

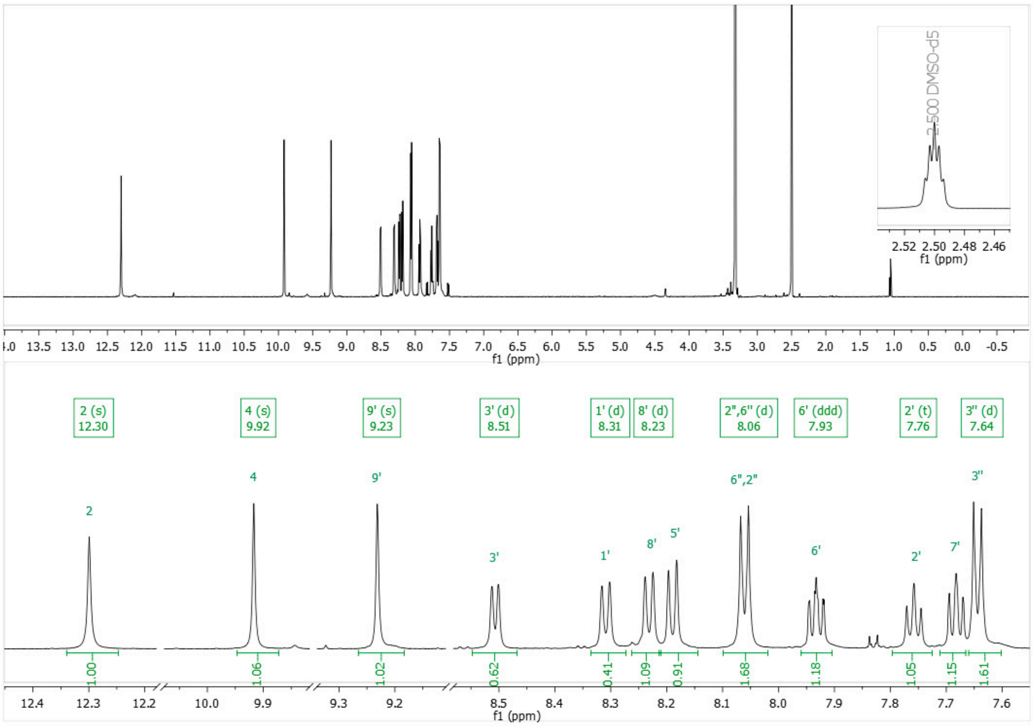

^{1}H NMR spectrum (600 MHz, DMSO-d$_6$) of the derivative **3c**.

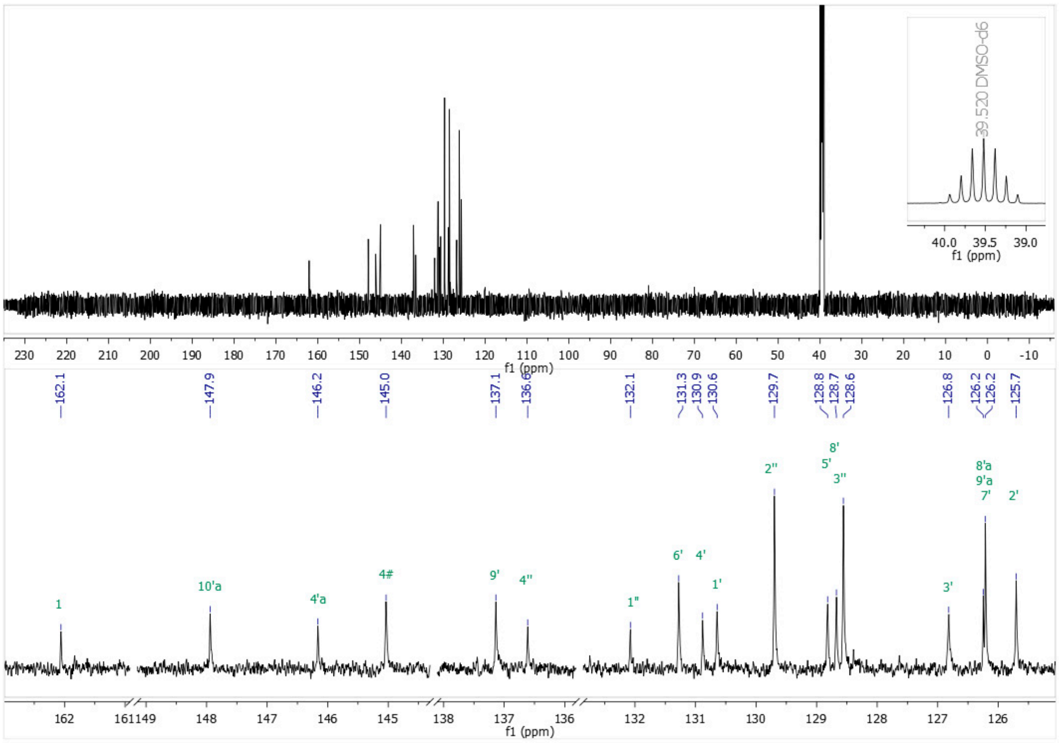

^{13}C NMR spectrum (151 MHz, DMSO-d$_6$) of the derivative **3c**.

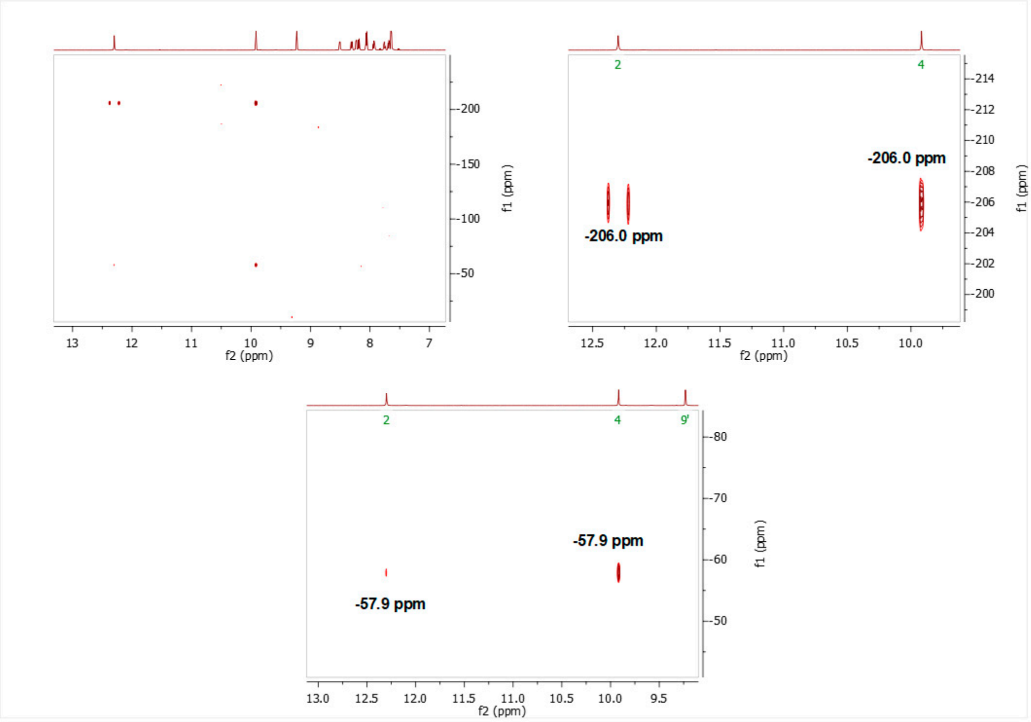

$^{1}H,^{15}N$-HMBC spectrum (600/61 MHz, DMSO-d_6) of the derivative **3c**.

1.4 *N'*-[(*E*)-Acridin-4-yl)methylidene]-4-bromobenzohydrazide (3d)

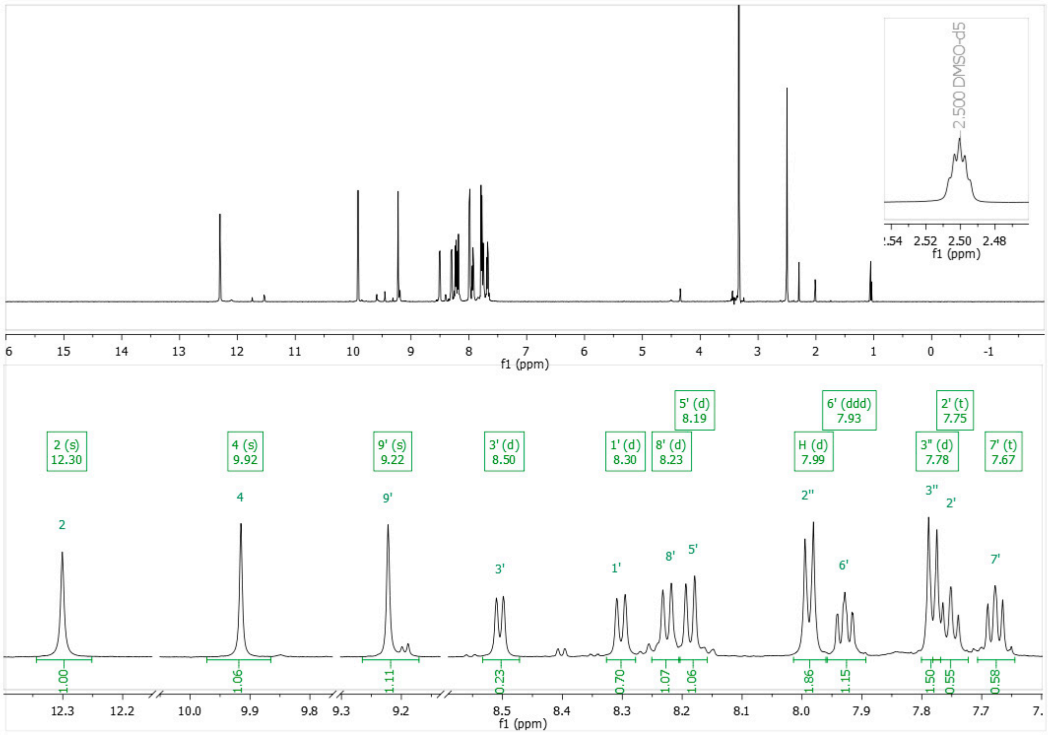

^{1}H NMR spectrum (600 MHz, DMSO-d$_6$) of the derivative **3d**.

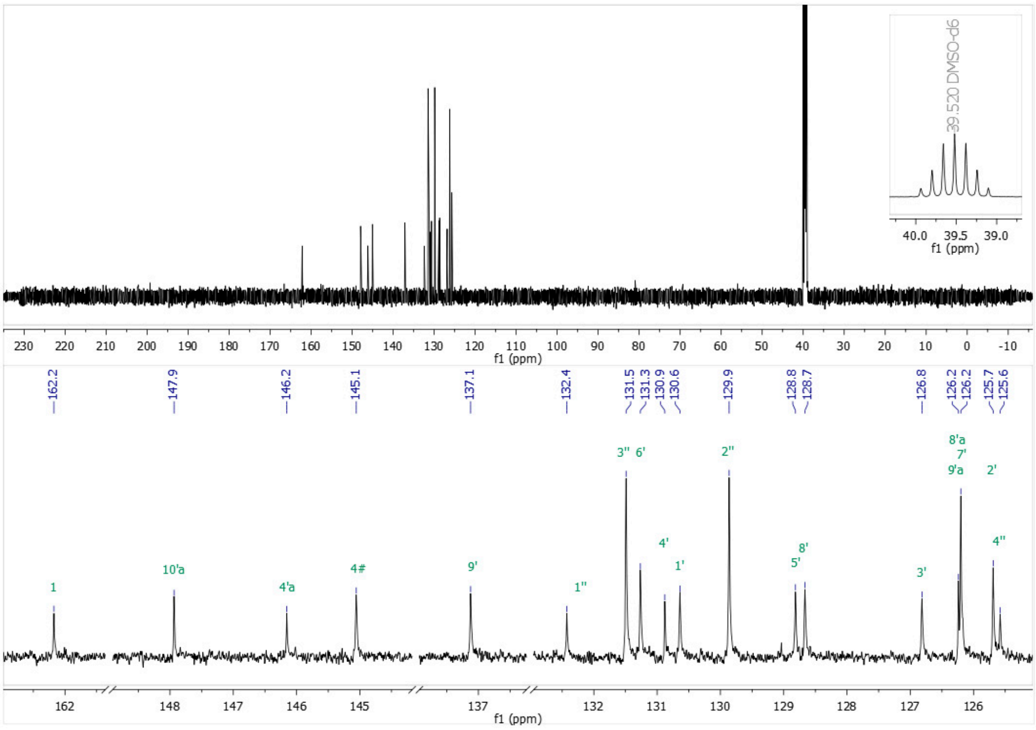

^{13}C NMR spectrum (151 MHz, DMSO-d$_6$) of the derivative **3d**.

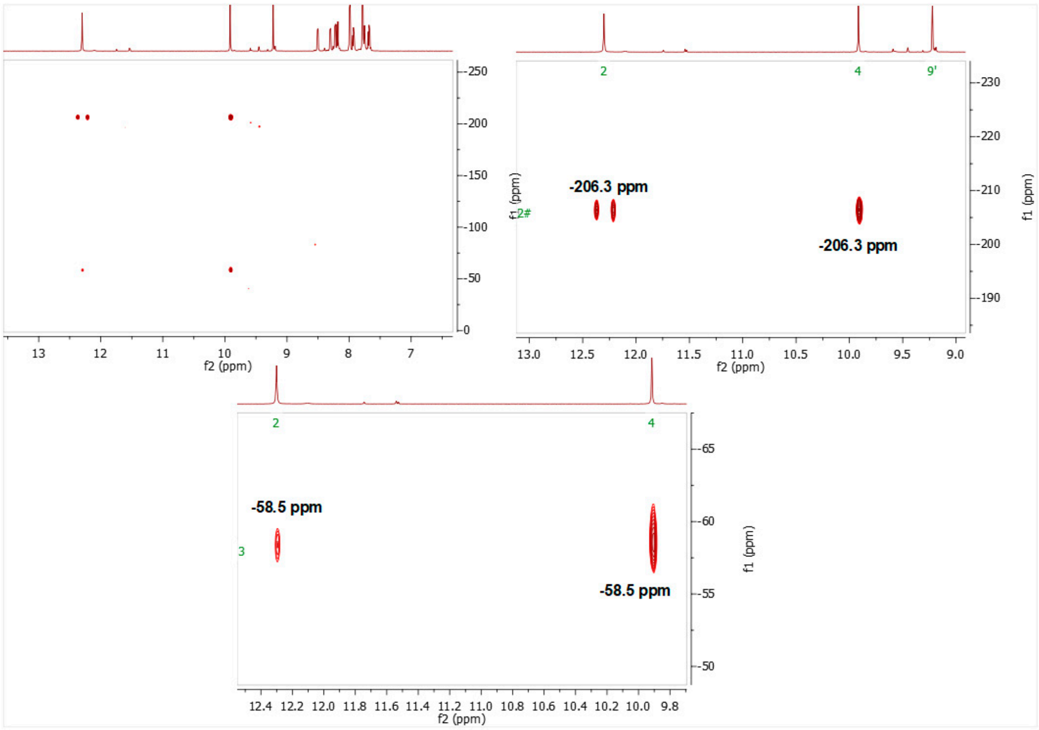

$^{1}H,^{15}N$-HMBC spectrum (600/61 MHz, DMSO-d_6) of the derivative **3d**.

1.5 *N'*-[(*E*)-Acridin-4-yl)methylidene]-4-methoxybenzohydrazide (3e)

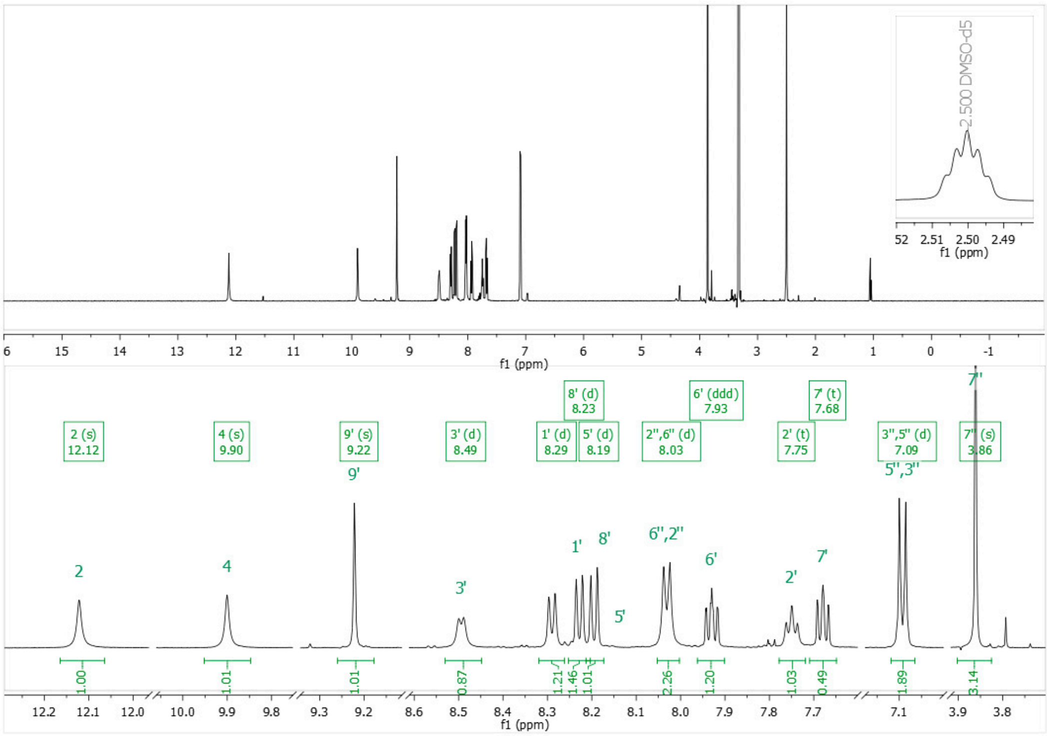

^{1}H NMR spectrum (600 MHz, DMSO-d$_6$) of the derivative **3e**.

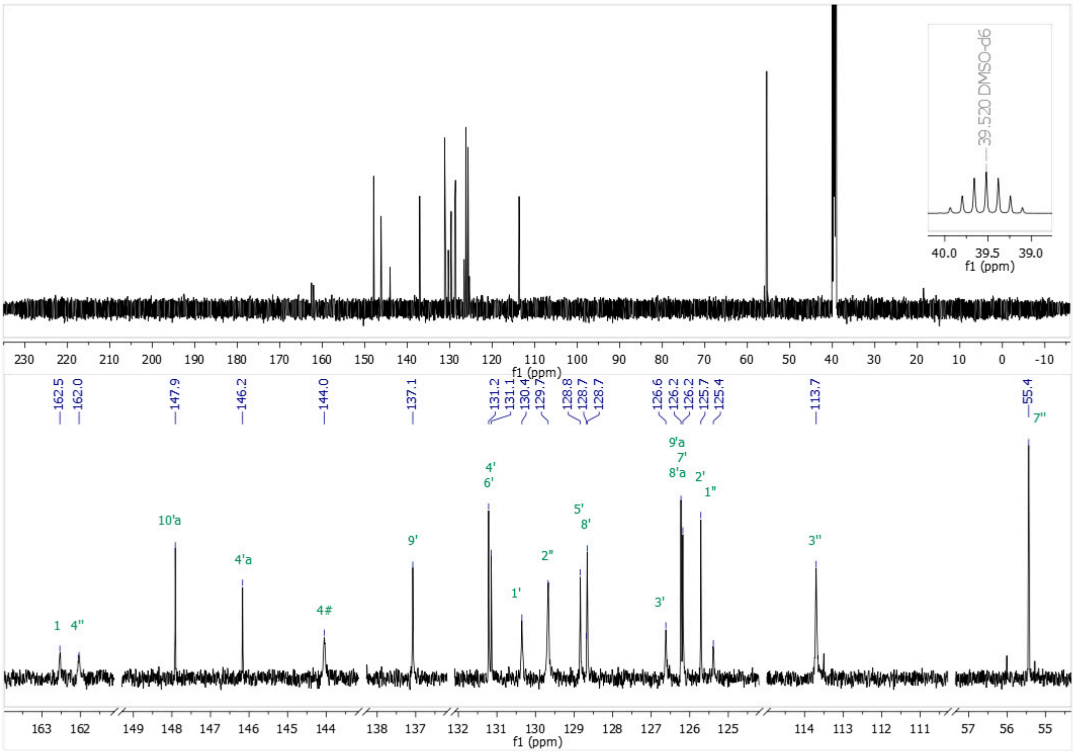

^{13}C NMR spectrum (151 MHz, DMSO-d$_6$) of the derivative **3e**.

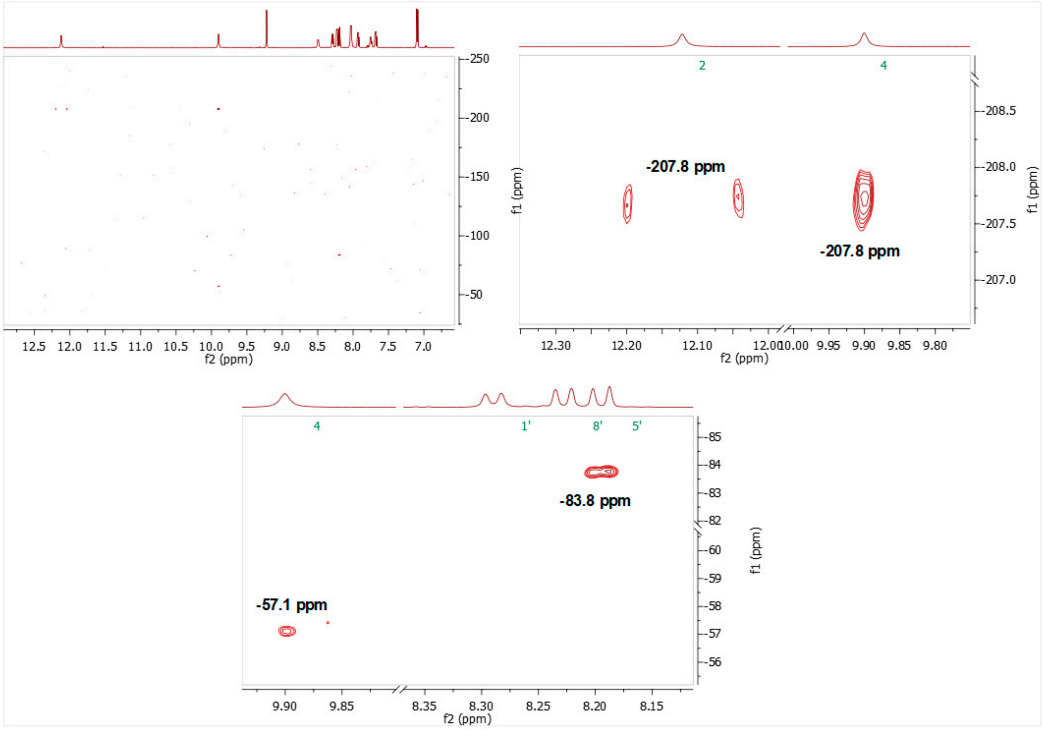

^{1}H,^{15}N-HMBC spectrum (600/61 MHz, DMSO-d$_6$) of the derivative **3e**.

1.6 *N*'-[(*E*)-4-fluorophenylmethylidene]acridine-4-carbohydrazide 7b

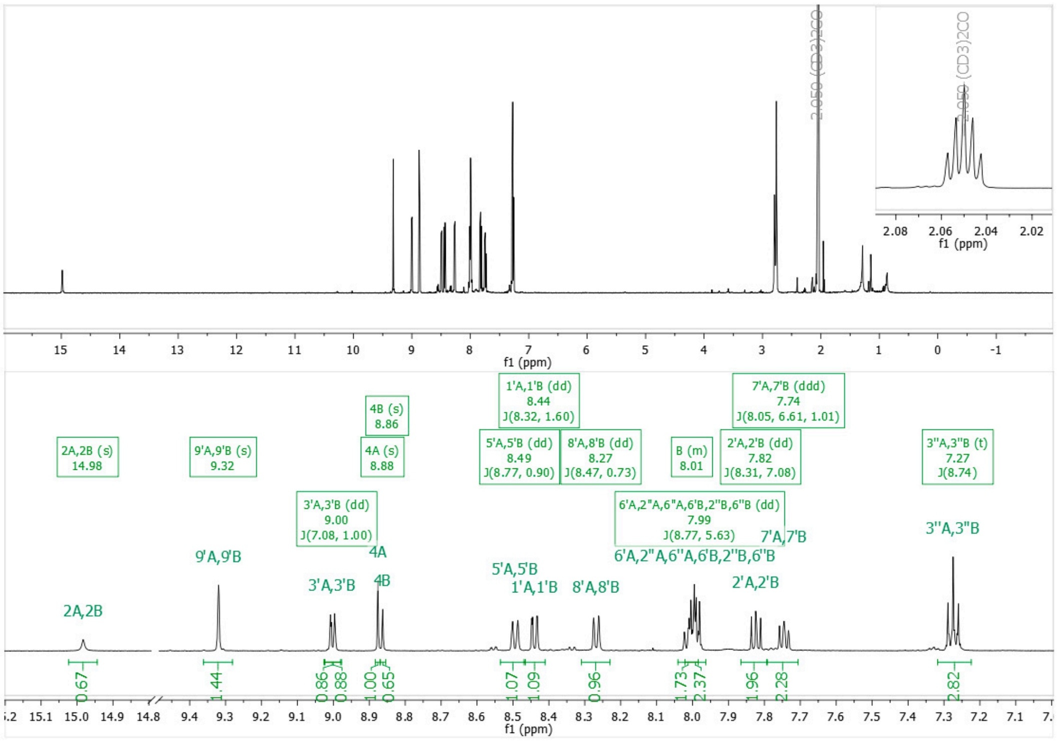

^{1}H NMR spectrum (600 MHz, DMSO-d$_6$) of the derivative **7b** (A: $E_{\mathrm{C=N}}Z_{\mathrm{C(O)-N}}Z_{\mathrm{N-N}}$, B: $E_{\mathrm{C=N}}Z_{\mathrm{C(O)-N}}E_{\mathrm{N-N}}$).

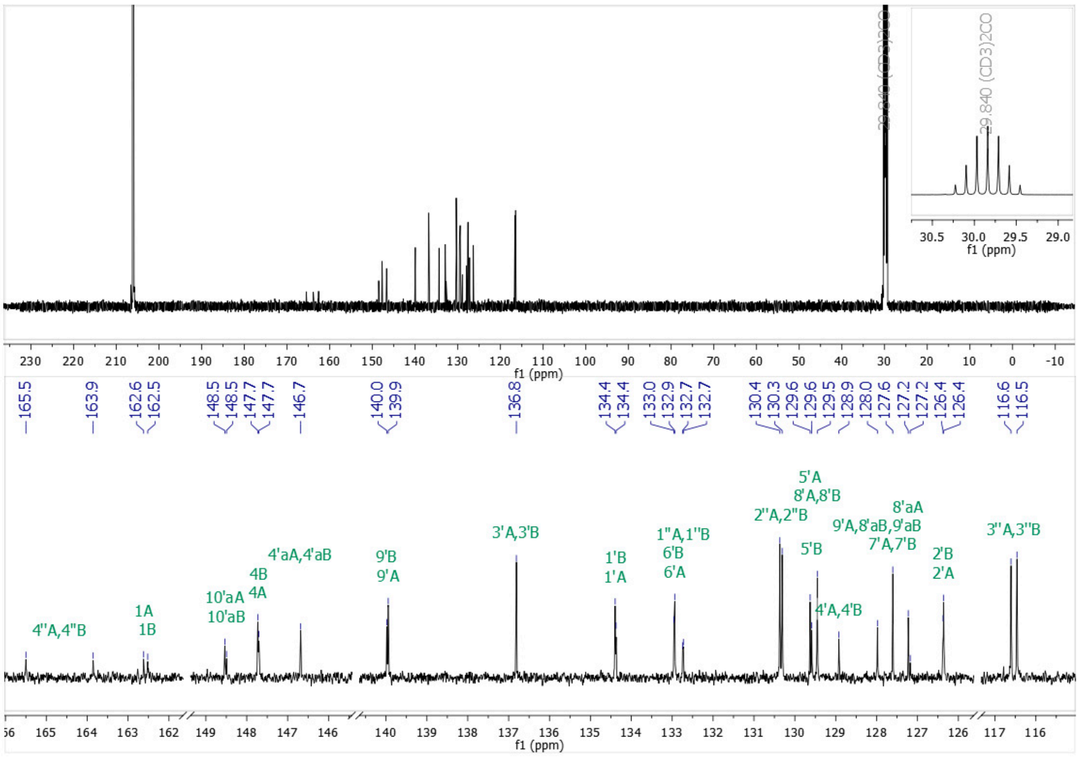

^{13}C NMR spectrum (151 MHz, DMSO-d$_6$) of the derivative **7b** (A: $E_{\mathrm{C=N}}Z_{\mathrm{C(O)-N}}Z_{\mathrm{N-N}}$, B: $E_{\mathrm{C=N}}Z_{\mathrm{C(O)-N}}E_{\mathrm{N-N}}$).

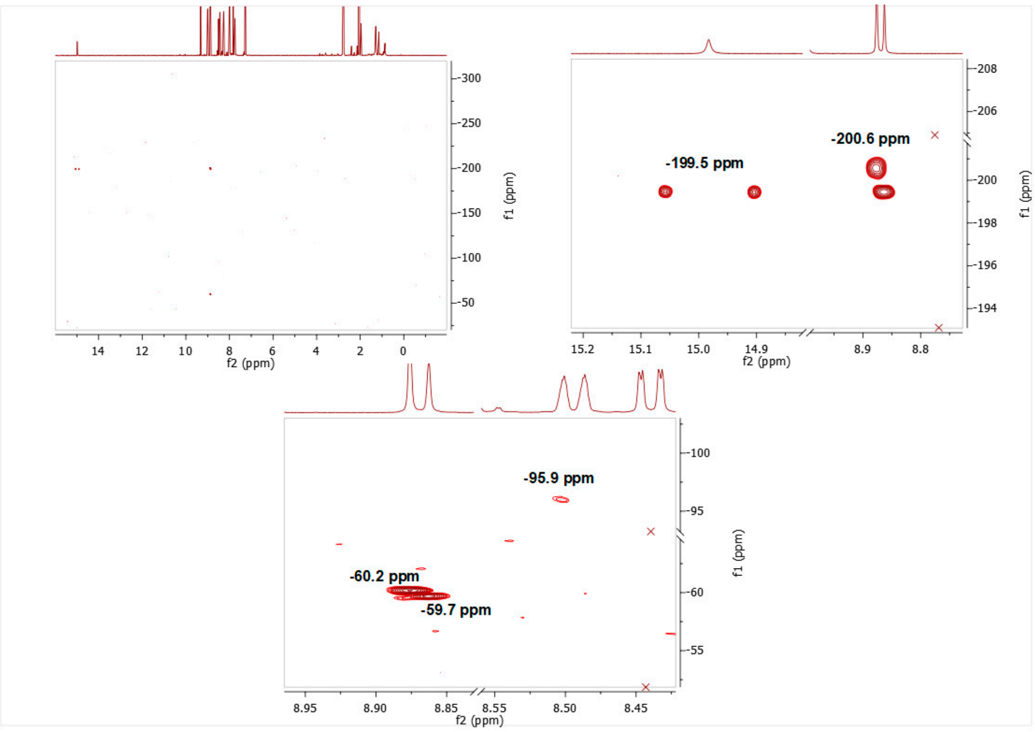

^{1}H,^{15}N-HMBC spectrum (600/61 MHz, DMSO-d$_6$) of the derivative **7b** (A: $E_{C=N}Z_{C(O)–N}Z_{N–N}$, B: $E_{C=N}Z_{C(O)–N}E_{N–N}$).

1.7 *N*'-[(*E*)-4-chlorophenylmethylidene]acridine-4-carbohydrazide 7c

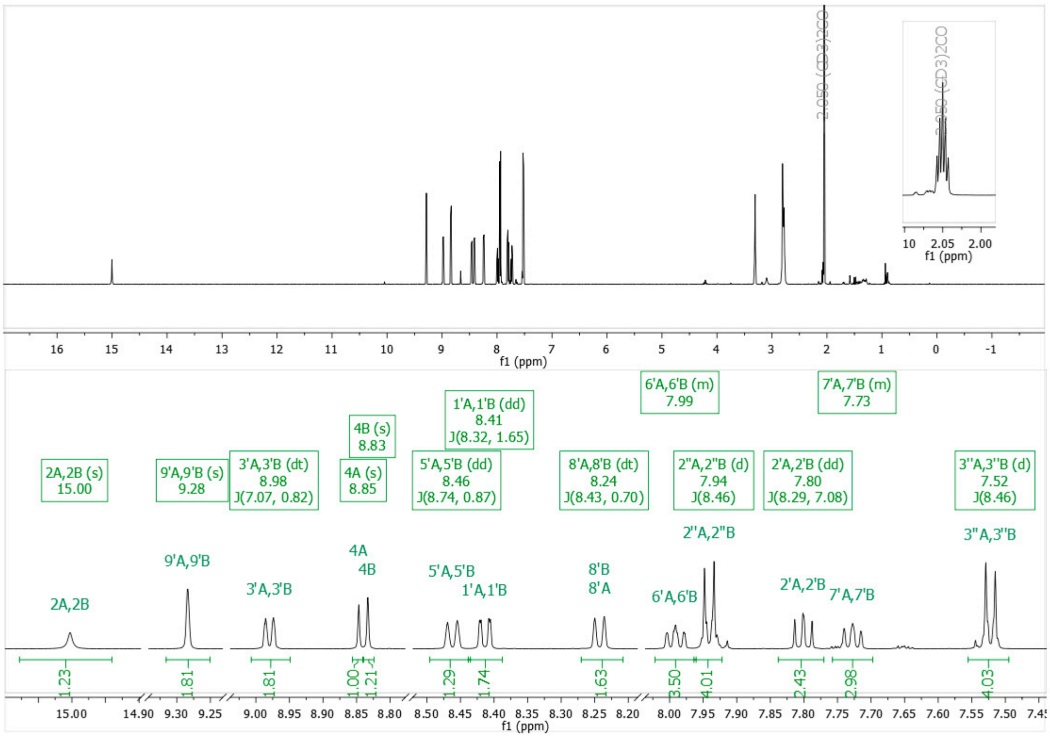

^{1}H NMR spectrum (600 MHz, DMSO-d6) of the derivative **7c** (A: $E_{C=N}Z_{C(O)-N}Z_{N-N}$, B: $E_{C=N}Z_{C(O)-N}E_{N-N}$).

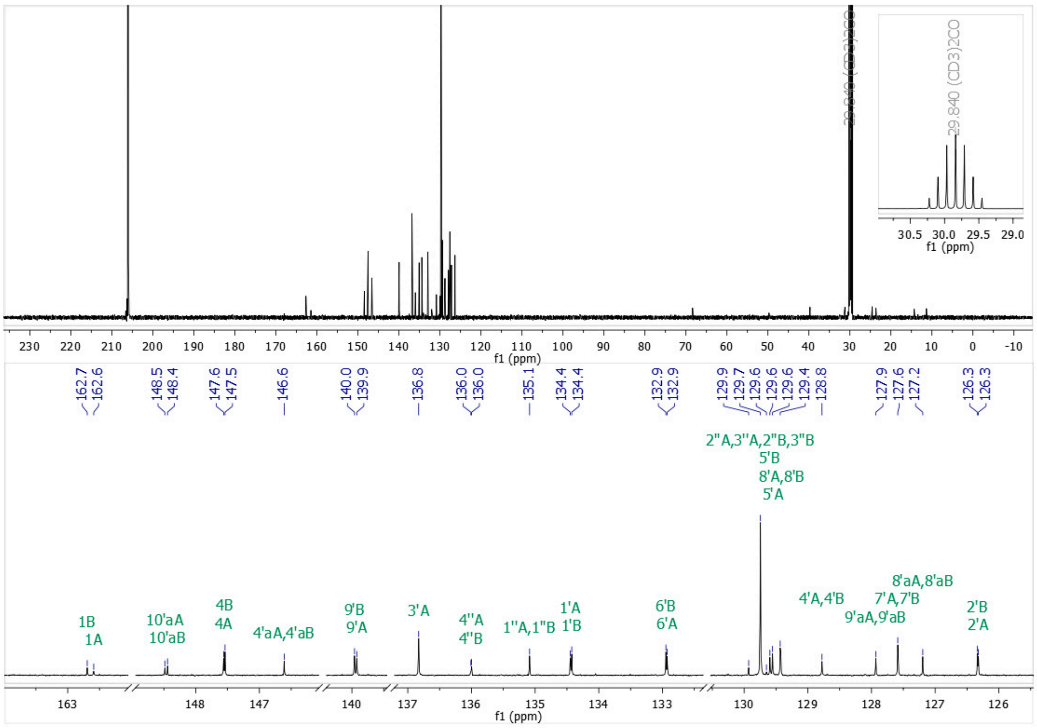

^{13}C NMR spectrum (151 MHz, DMSO-d6) of the derivative **7c** (A: $E_{C=N}Z_{C(O)-N}Z_{N-N}$, B: $E_{C=N}Z_{C(O)-N}E_{N-N}$).

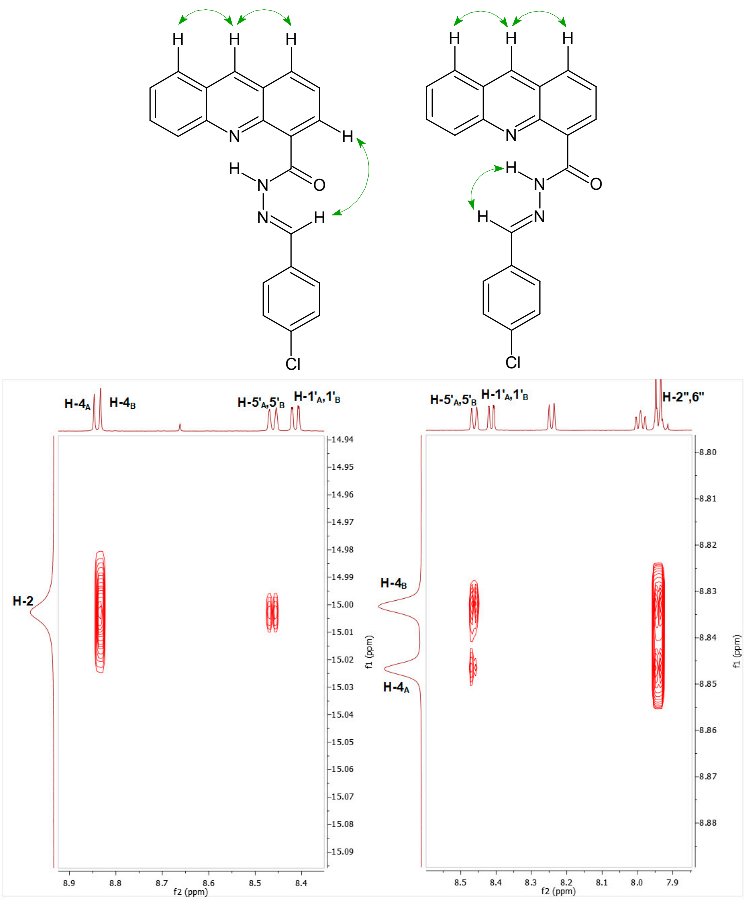

The selected areas of 2D NOESY spectra of derivative $E_{C=N}Z_{C(O)-N}$-**7c** showing key NOESY cross peaks.

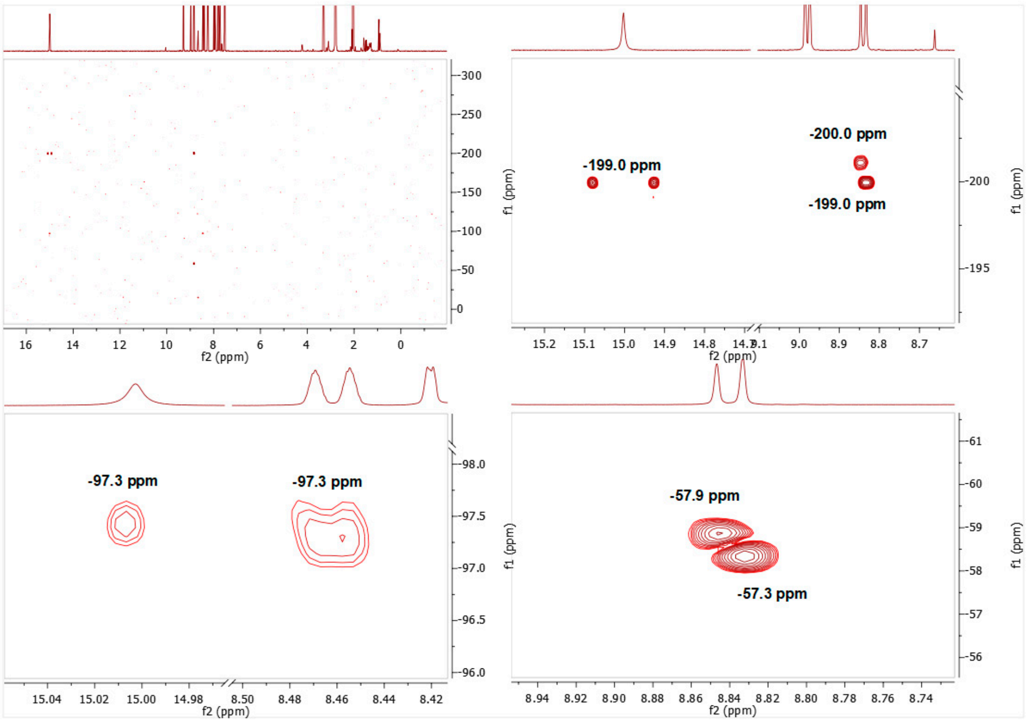

$^{1}H,^{15}N$-HMBC spectrum (600/61 MHz, DMSO-d_6) of the derivative **7c** (A: $E_{C=N}Z_{C(O)-N}Z_{N-N}$, B: $E_{C=N}Z_{C(O)-N}E_{N-N}$).

1.8 *N'*-[(*E*)-4-bromophenylmethylidene]acridine-4-carbohydrazide 7d

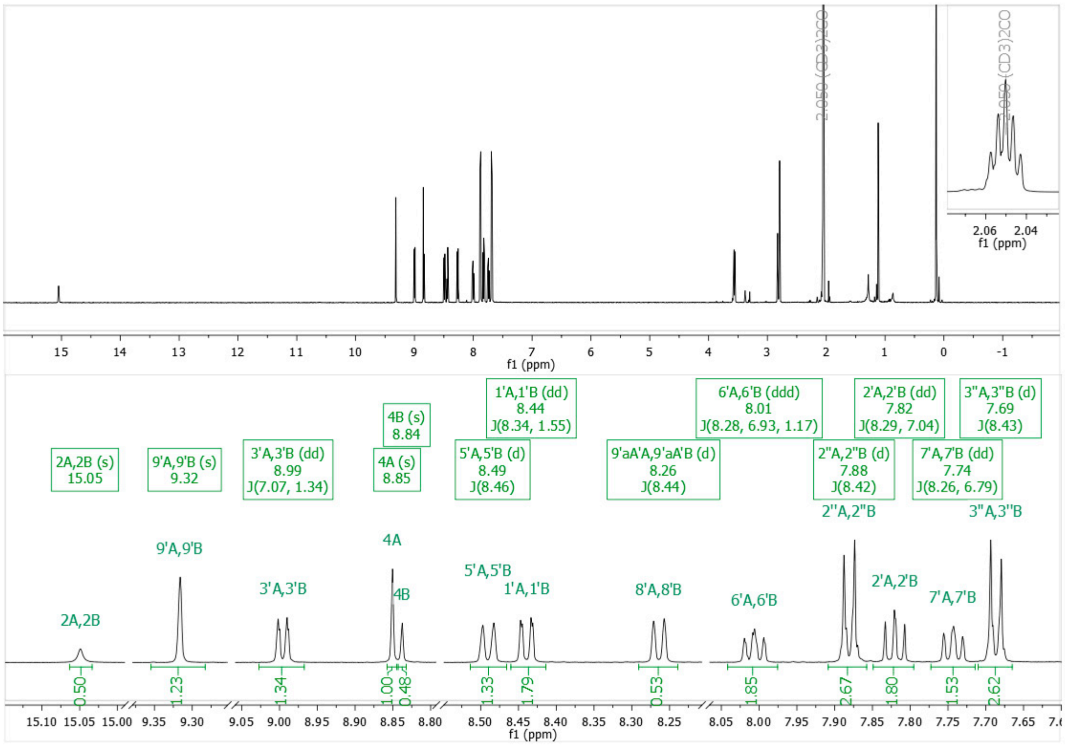

^{1}H NMR spectrum (600 MHz, DMSO-d$_6$) of the derivative **7d** (A: $E_{C=N}Z_{C(O)-N}Z_{N-N}$, B: $E_{C=N}Z_{C(O)-N}E_{N-N}$).

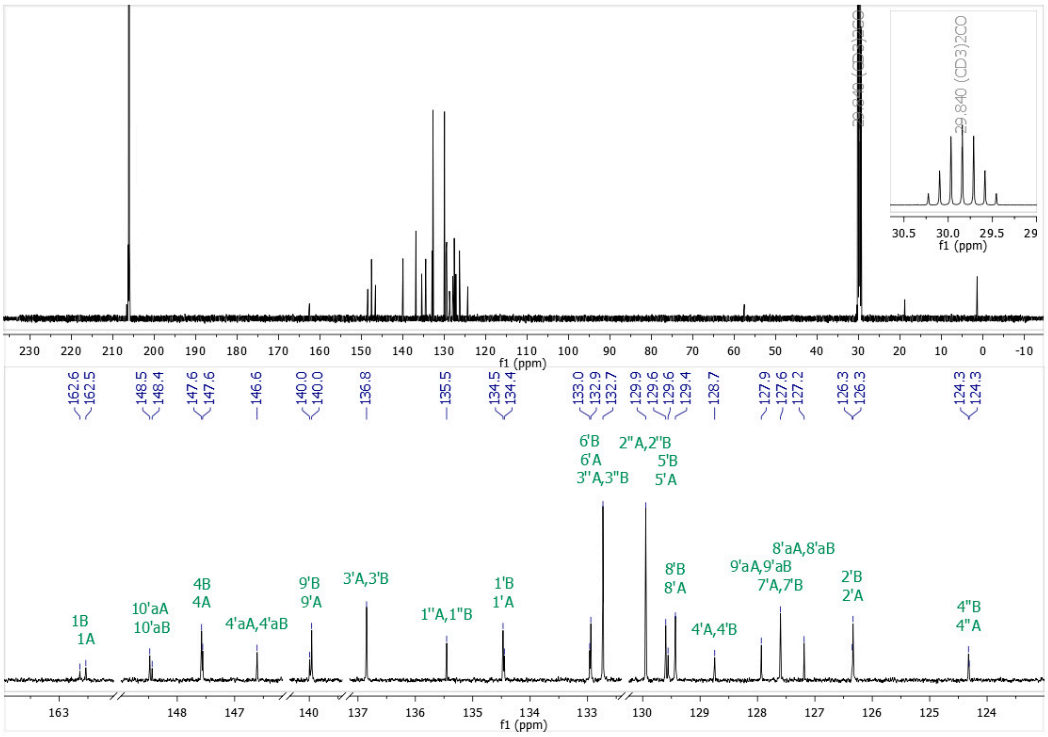

^{13}C NMR spectrum (151 MHz, DMSO-d$_6$) of the derivative **7d** (A: $E_{C=N}Z_{C(O)-N}Z_{N-N}$, B: $E_{C=N}Z_{C(O)-N}E_{N-N}$).

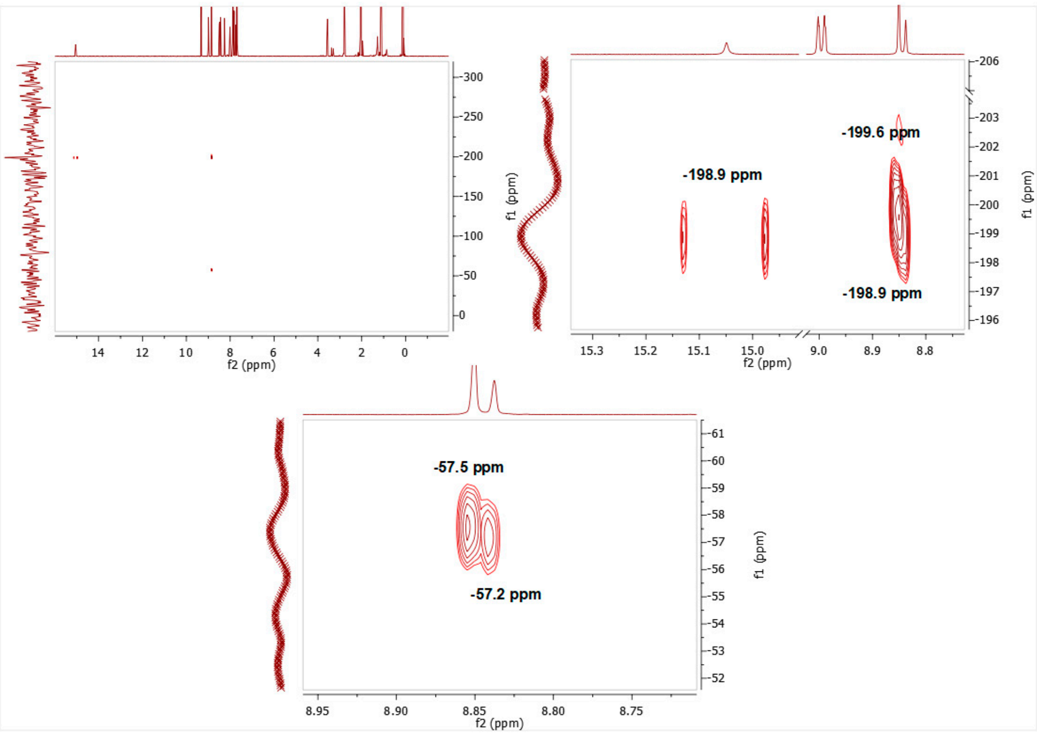

^{1}H,^{15}N-HMBC spectrum (600/61 MHz, DMSO-d6) of the derivative **7d** (A: $E_{C=N}Z_{C(O)-N}Z_{N-N}$, B: $E_{C=N}Z_{C(O)-N}E_{N-N}$).

3 HR MS spectra of derivatives 3a–e and 7b–d

1.9 *N*'-[(*E*)-Acridin-4-yl)methylidene]benzohydrazide (3a)

Single Mass Analysis
Tolerance = 2.0 PPM / DBE: min = -1.5, max = 50.0
Element prediction: Off
Number of isotope peaks used for i-FIT = 3
Monoisotopic Mass, Even Electron Ions
416 formula(e) evaluated with 1 results within limits (up to 50 best isotopic matches for each mass)
Elements Used:

Mass	Calc. Mass	mDa	PPM	DBE	Formula	i-FIT	i-FIT Norm	Fit Conf %	C	H	N	O
326.1291	326.1293	-0.2	-0.6	15.5	C21 H16 N3 O	1006.4	n/a	n/a	21	16	3	1

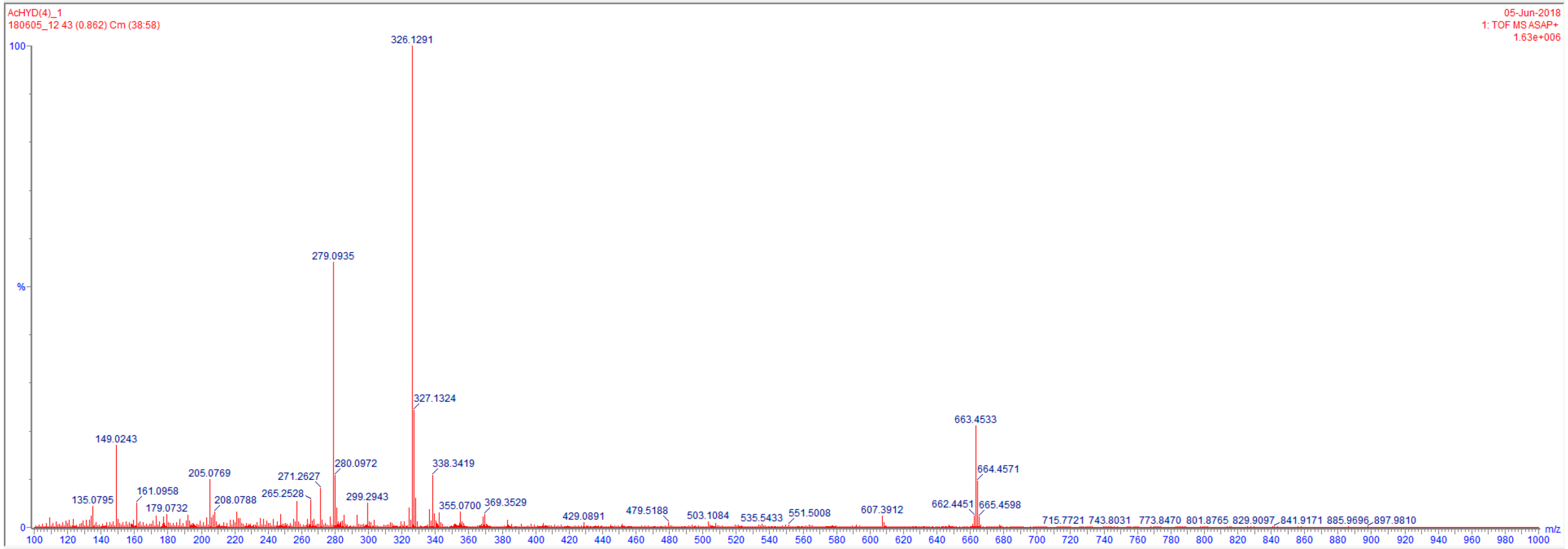

For Help, press F1

1.10 *N*'-[(*E*)-Acridin-4-yl)methylidene]-4-fluorobenzohydrazide (3b)

Single Mass Analysis
Tolerance = 2.0 PPM / DBE: min = -1.5, max = 50.0
Element prediction: Off
Number of isotope peaks used for i-FIT = 3
Monoisotopic Mass, Even Electron Ions
416 formula(e) evaluated with 1 results within limits (up to 50 best isotopic matches for each mass)
Elements Used:

Mass	Calc. Mass	mDa	PPM	DBE	Formula	i-FIT	i-FIT Norm	Fit Conf %	C	H	N	O	F
344.1201	344.1199	0.2	0.6	15.5	C21 H15 N3 O F	995.9	n/a	n/a	21	15	3	1	1

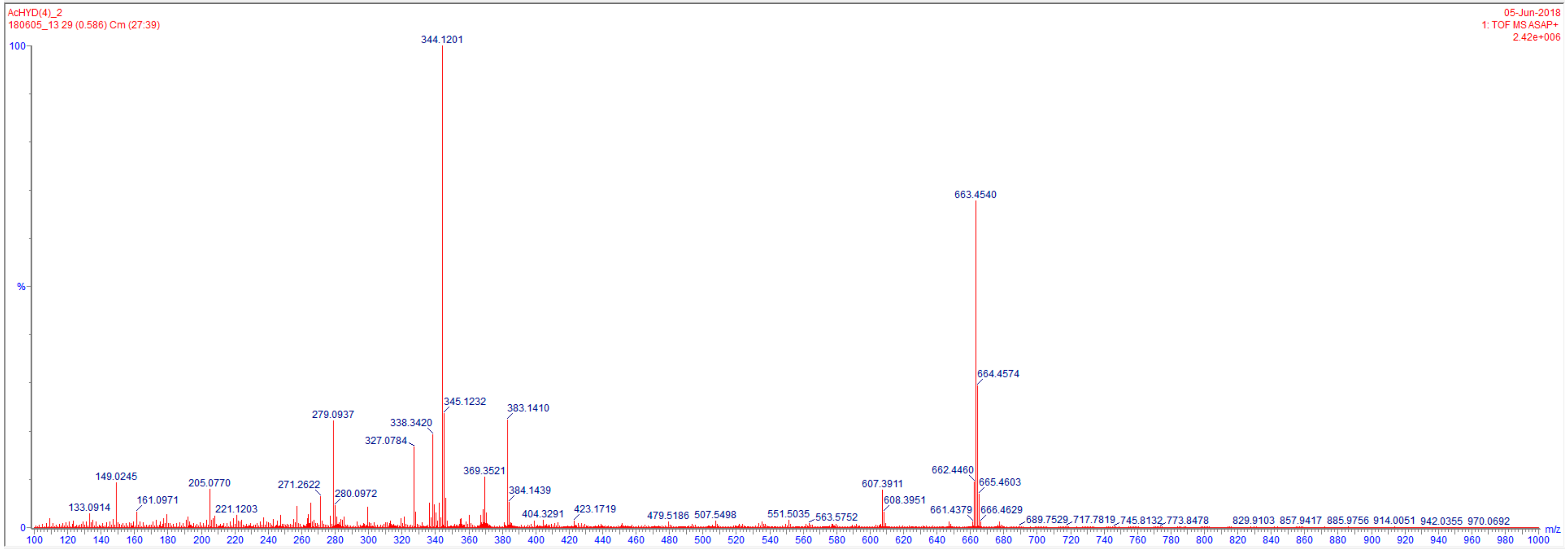

For Help, press F1

1.11 *N'*-[(*E*)-Acridin-4-yl)methylidene]-4-chlorobenzohydrazide (3c)

Single Mass Analysis
Tolerance = 2.0 PPM / DBE: min = -1.5, max = 50.0
Element prediction: Off
Number of isotope peaks used for i-FIT = 3
Monoisotopic Mass, Even Electron Ions
416 formula(e) evaluated with 1 results within limits (up to 50 best isotopic matches for each mass)
Elements Used:

Mass	Calc. Mass	mDa	PPM	DBE	Formula	i-FIT	i-FIT Norm	Fit Conf %	C	H	N	O	Cl
360.0905	360.0904	0.1	0.3	15.5	C21 H15 N3 O Cl	1033.1	n/a	n/a	21	15	3	1	1

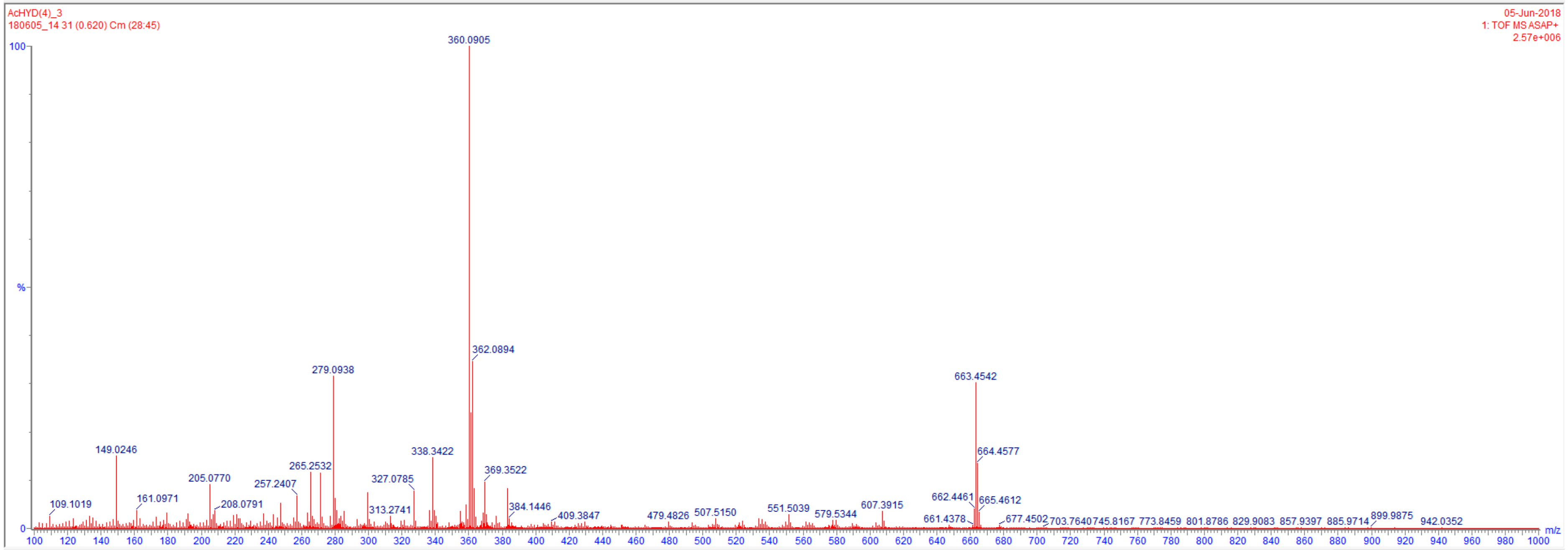

For Help, press F1

1.12 *N*'-[(*E*)-Acridin-4-yl)methylidene]-4-bromobenzohydrazide (3d)

Single Mass Analysis
Tolerance = 2.0 PPM / DBE: min = -1.5, max = 50.0
Element prediction: Off
Number of isotope peaks used for i-FIT = 3
Monoisotopic Mass, Even Electron Ions
416 formula(e) evaluated with 1 results within limits (up to 50 best isotopic matches for each mass)
Elements Used:

Mass	Calc. Mass	mDa	PPM	DBE	Formula	i-FIT	i-FIT Norm	Fit Conf %	C	H	N	O	Br
404.0396	404.0399	-0.3	-0.7	15.5	C21 H15 N3 O Br	1278.9	n/a	n/a	21	15	3	1	1

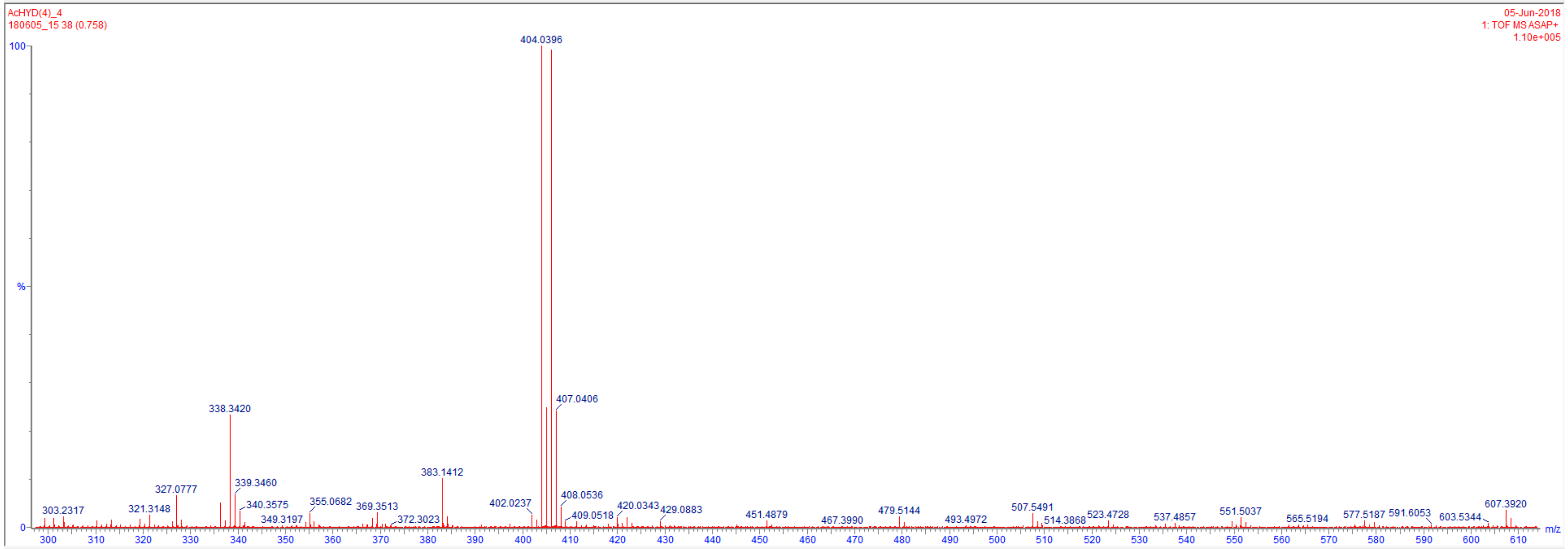

For Help, press F1

1.13 *N*'-[(*E*)-Acridin-4-yl)methylidene]-4-methoxybenzohydrazide (3e)

Single Mass Analysis
Tolerance = 2.0 PPM / DBE: min = -1.5, max = 50.0
Element prediction: Off
Number of isotope peaks used for i-FIT = 3
Monoisotopic Mass, Even Electron Ions
460 formula(e) evaluated with 1 results within limits (up to 50 best isotopic matches for each mass)
Elements Used:

Mass	Calc. Mass	mDa	PPM	DBE	Formula	i-FIT	i-FIT Norm	Fit Conf %	C	H	N	O
356.1398	356.1399	-0.1	-0.3	15.5	C22 H18 N3 O2	1011.4	n/a	n/a	22	18	3	2

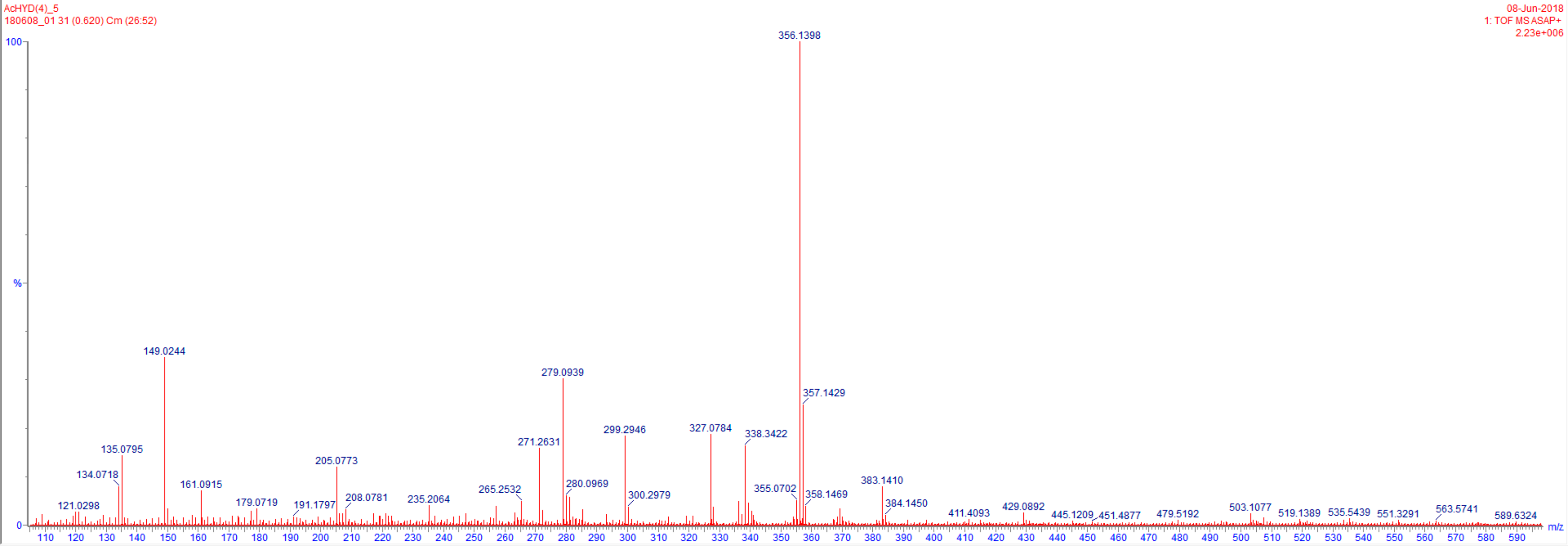

For Help, press F1

1.14 *N'*-[(*E*)-4-Fluorophenylmethylidene]acridine-4-carbohydrazide (7b)

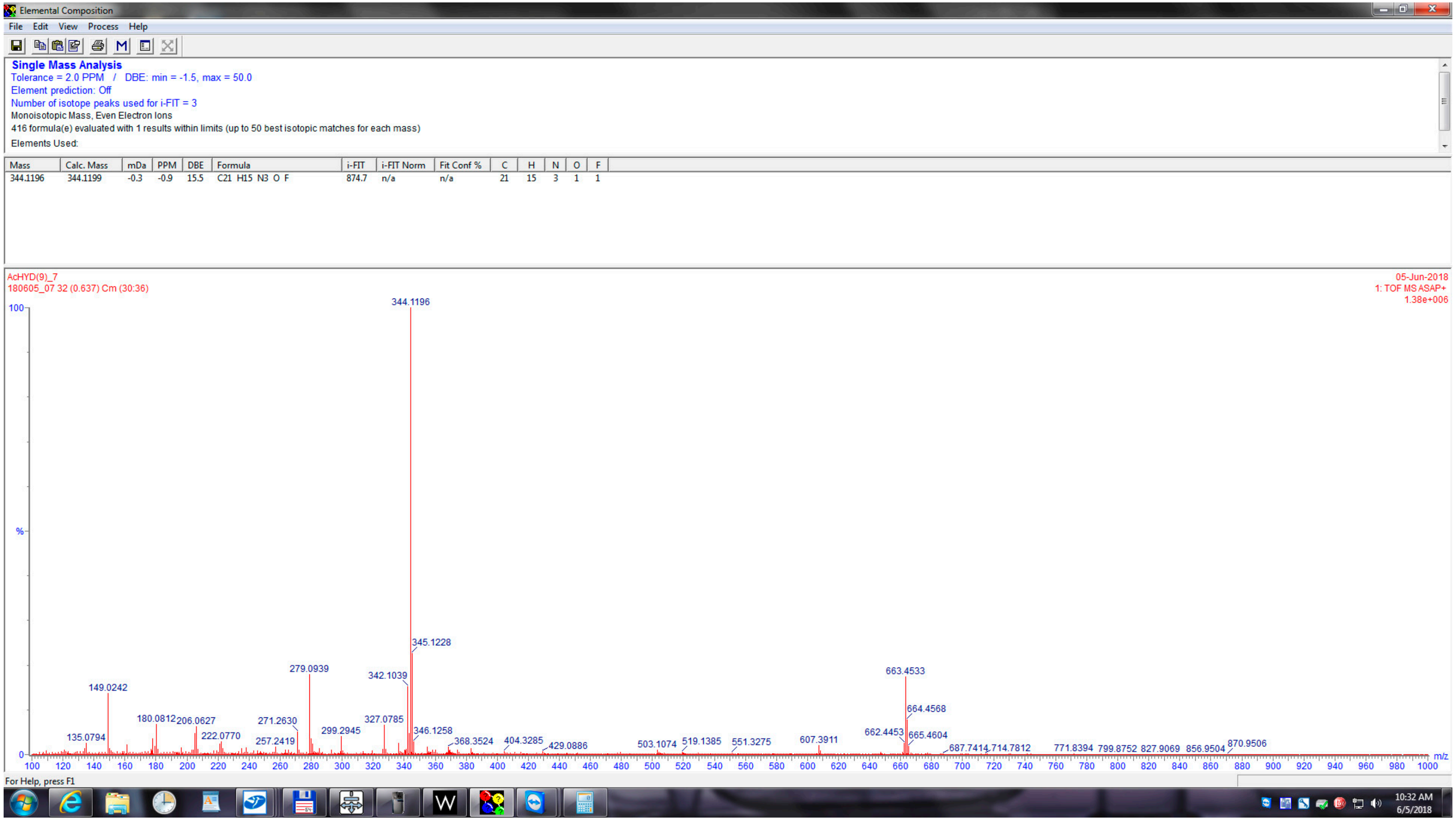

1.15 *N'*-[(*E*)-4-Chlorophenylmethylidene]acridine-4-carbohydrazide (7c)

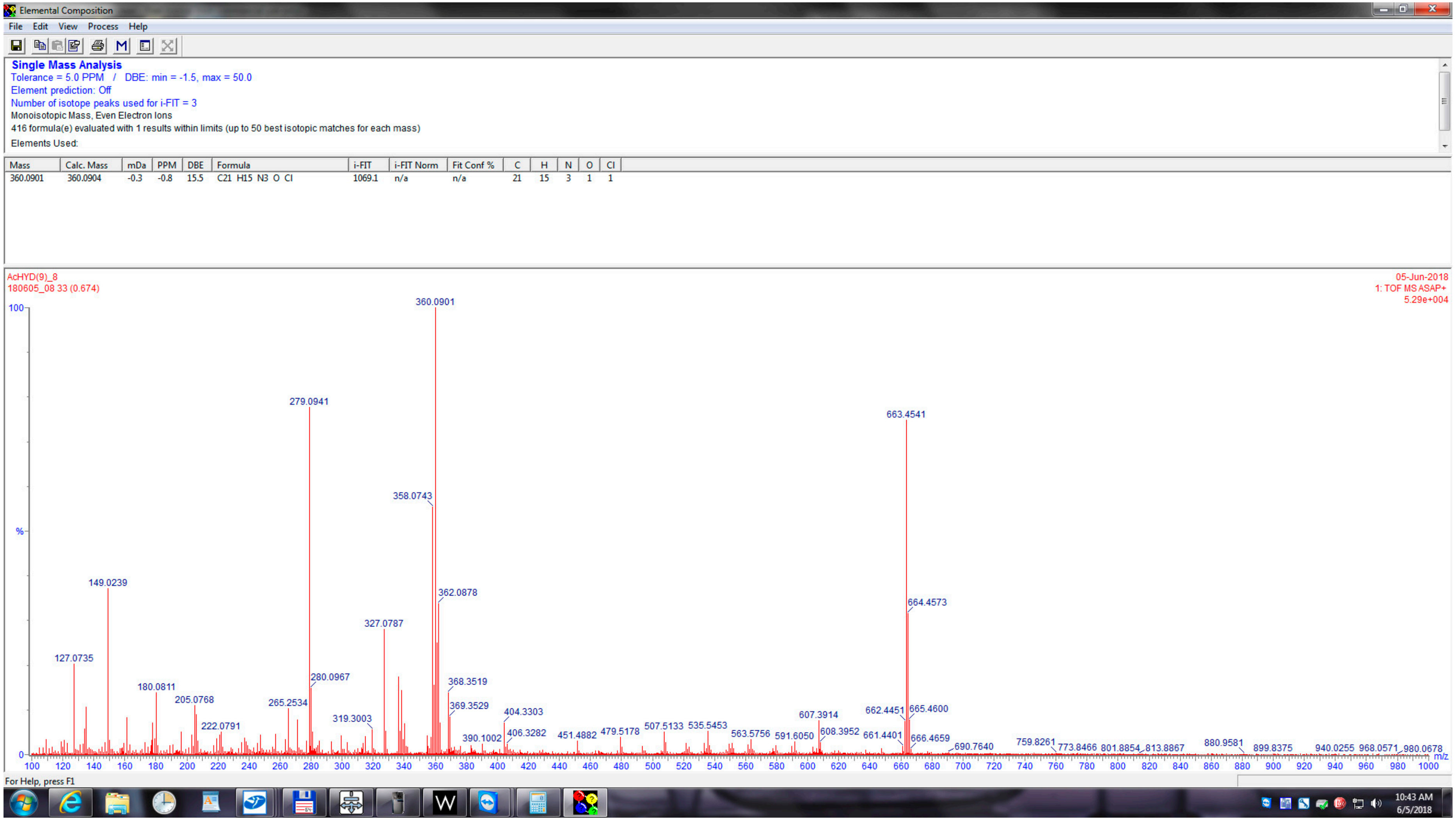

1.16 *N'*-[(*E*)-4-Bromophenylmethylidene]acridine-4-carbohydrazide (7d)

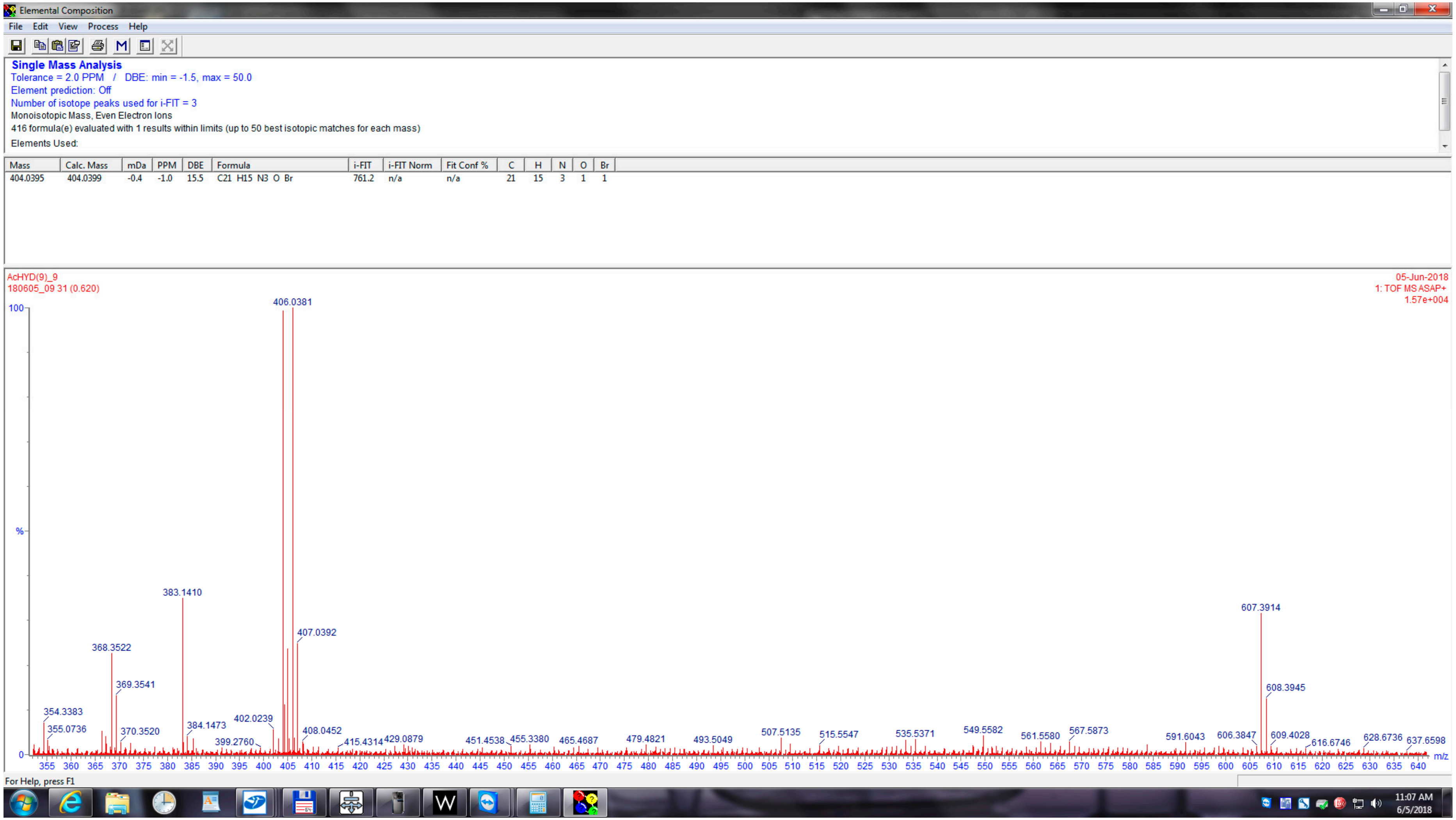

4 IR spectra of compounds 3a–d and 7b, 7d

Table S9. Assignment of characteristic IR absorption bands with corresponding wavenumbers (in cm^{-1}) and intensity.

	ν(NH)	*ν*(CH)$_{ar}$	*ν*(CH)$_{aliph}$	*ν*(C=O)	*ν*(C=N)	*ν*(CC)$_{ar}$	δ(CCH)$_{ar}$	γ(CCH)$_{ar}$
**3a**	3196<br>(w, br)	3058(w)<br>3026(w)	X	1651(s)	1623(w)	1589(w)<br>1568(w)<br>1515(w)	1013(s)	760(s)
**3b**	3190<br>(w, br)	3064(w)<br>3041(w)	X	1655(s)	1626(w)	1602(s)<br>1563(w)<br>1506(s)	1012(s)	757(s)
**3c**	3194<br>(w, br)	3084(w)<br>3060(w)<br>3037(w)	X	1652(s)	1625(w)	1595(m)<br>1570(w)<br>1516(s)	1014(s)	754(s)
**3d**	3195<br>(w, br)	3082(w)<br>3058(w)<br>3038(w)	X	1653(s)	1626(w)	1591(m)<br>1568(w)<br>1517(s)	1011(s)	754(s)
**3e**	3209<br>(w, br)	3062(w)<br>3040(w)<br>3010(w)	2939(w)<br>2841(w)	1645(s)	1627(w)	1603(s)<br>1575(w)<br>1509(s)	1017(s)	756(s)
**7b**	3369<br>(m, br)	3077(w)<br>3062(w)<br>3050(w)<br>3013(w)	X	1645(s)	N	1603(s)<br>1507(m)	1024(m)	749(s)
**7d**	3201<br>(w, br)	3089(w)<br>3052(w)	X	1655(s)	1621(m)	1587(m)<br>1561(m)<br>1518(s)	1011(m)	737(s)

ar - aromatic, aliph - aliphatic, w - weak, m - medium, s - strong, br - broad, N - not observed, X - the bond is not present in the compound

1.17 *N*'-[(*E*)-Acridin-4-yl)methylidene]benzohydrazide (3a)

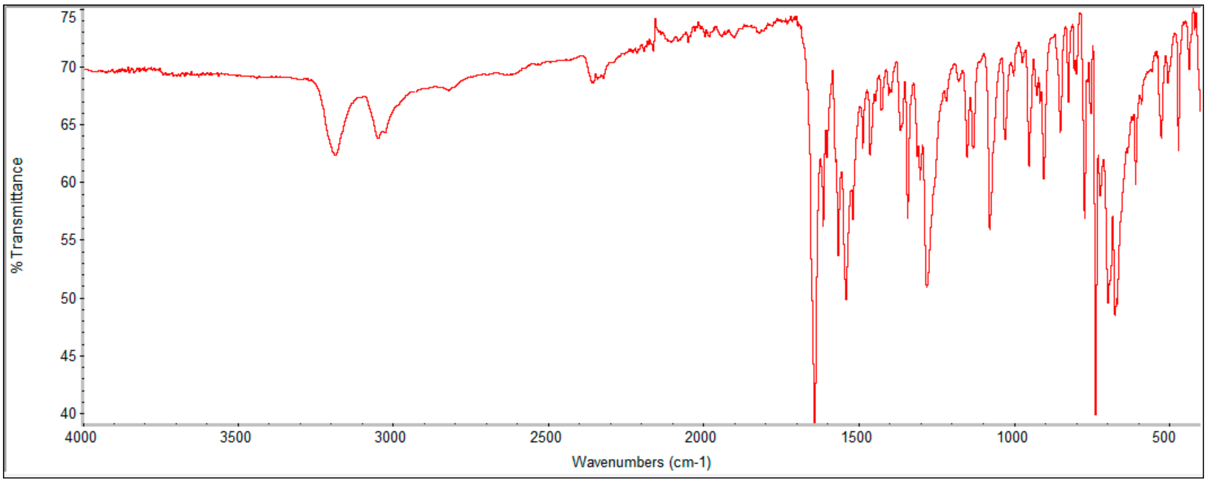

1.18 *N*'-[(*E*)-Acridin-4-yl)methylidene]-4-fluorobenzohydrazide (3b)

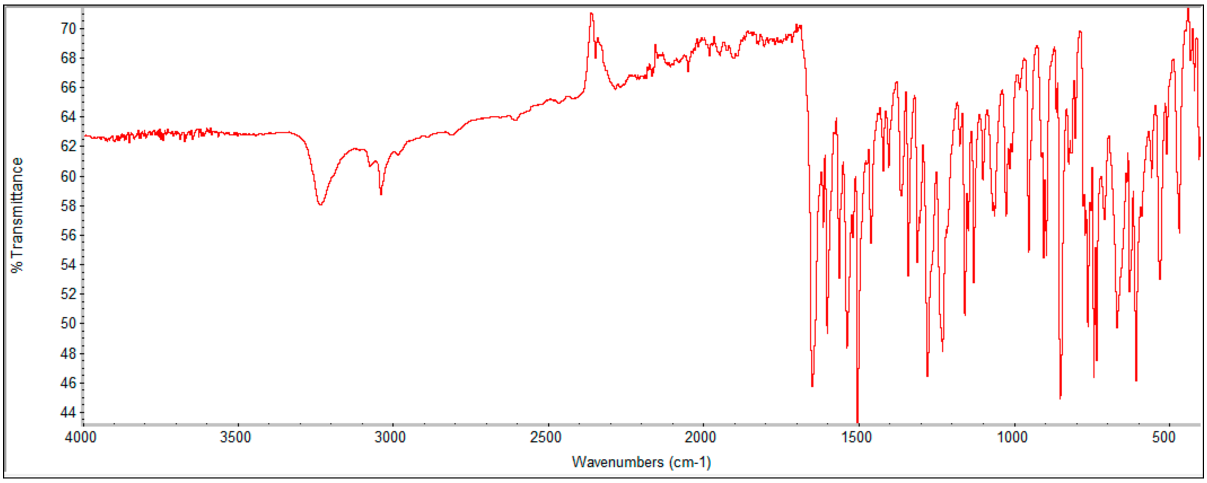

1.19 *N*'-[(*E*)-Acridin-4-yl)methylidene]-4-chlorobenzohydrazide (3c)

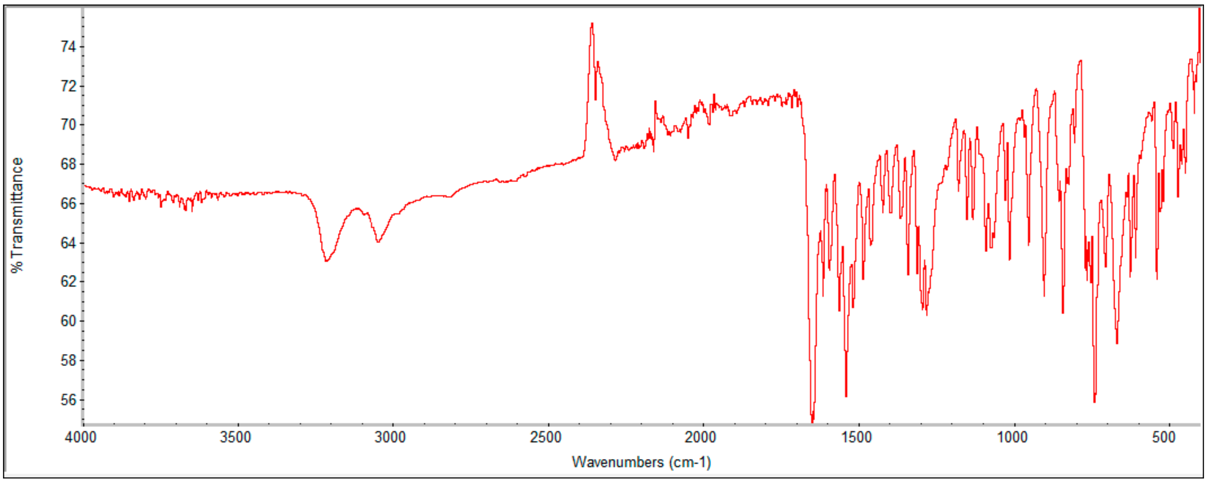

1.20 *N*'-[(*E*)-Acridin-4-yl)methylidene]-4-bromobenzohydrazide (3d)

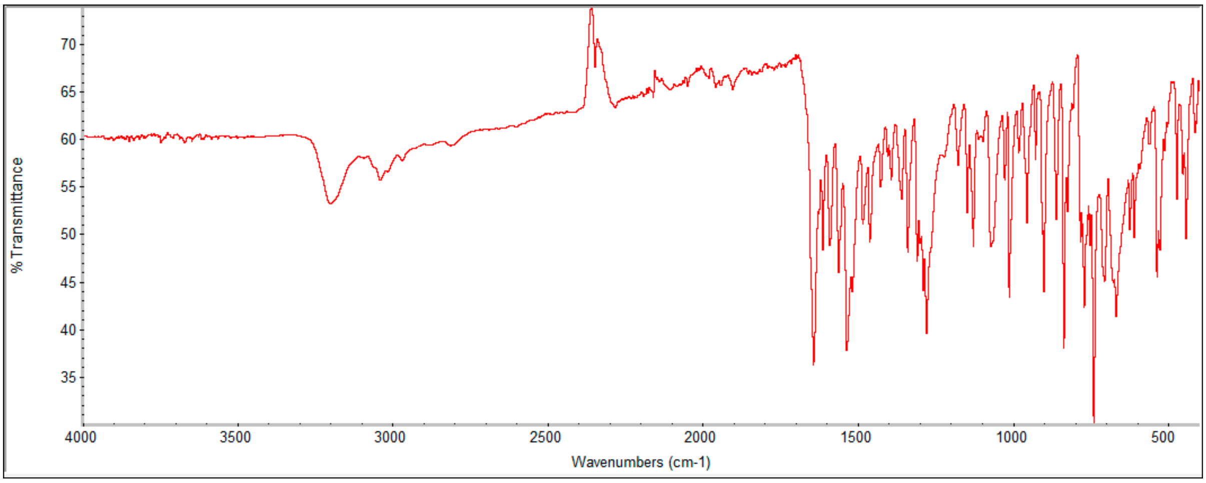

1.21 *N*'-[(*E*)-4-Fluorophenylmethylidene]acridine-4-carbohydrazide (7b)

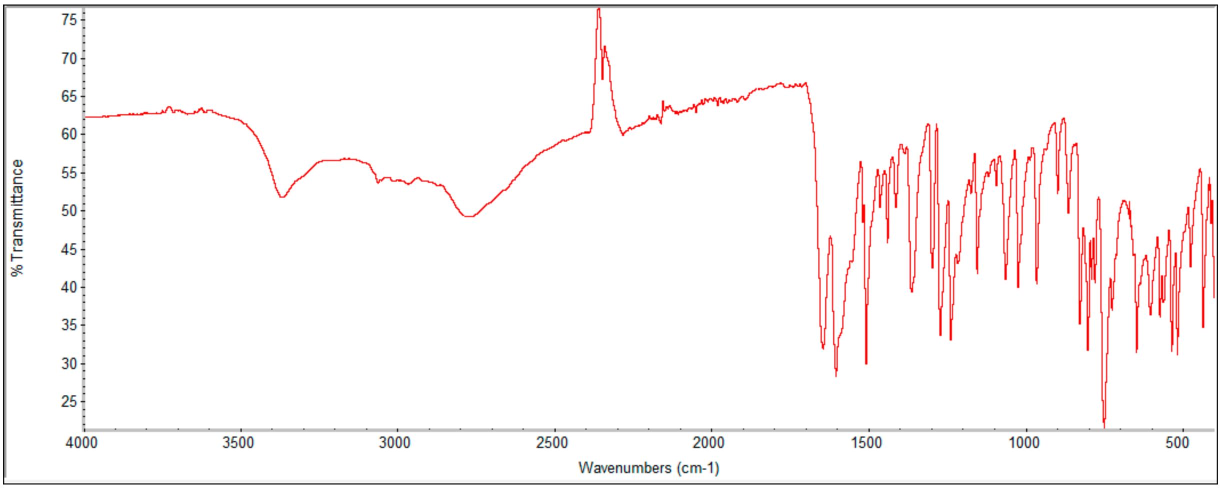

1.22 *N*'-[(*E*)-4-Bromophenylmethylidene]acridine-4-carbohydrazide (7d)

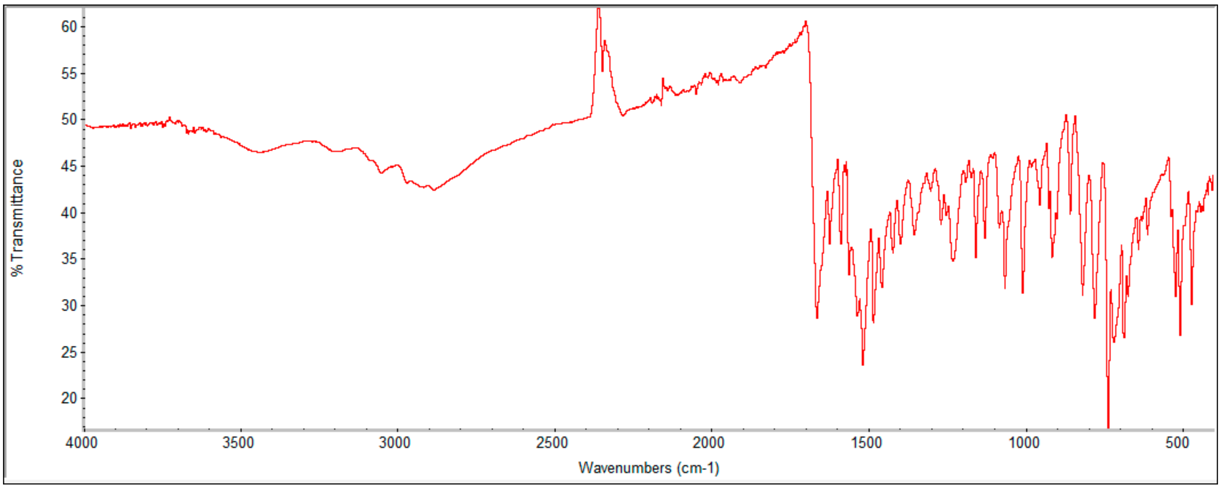

5 HSA binding experiments

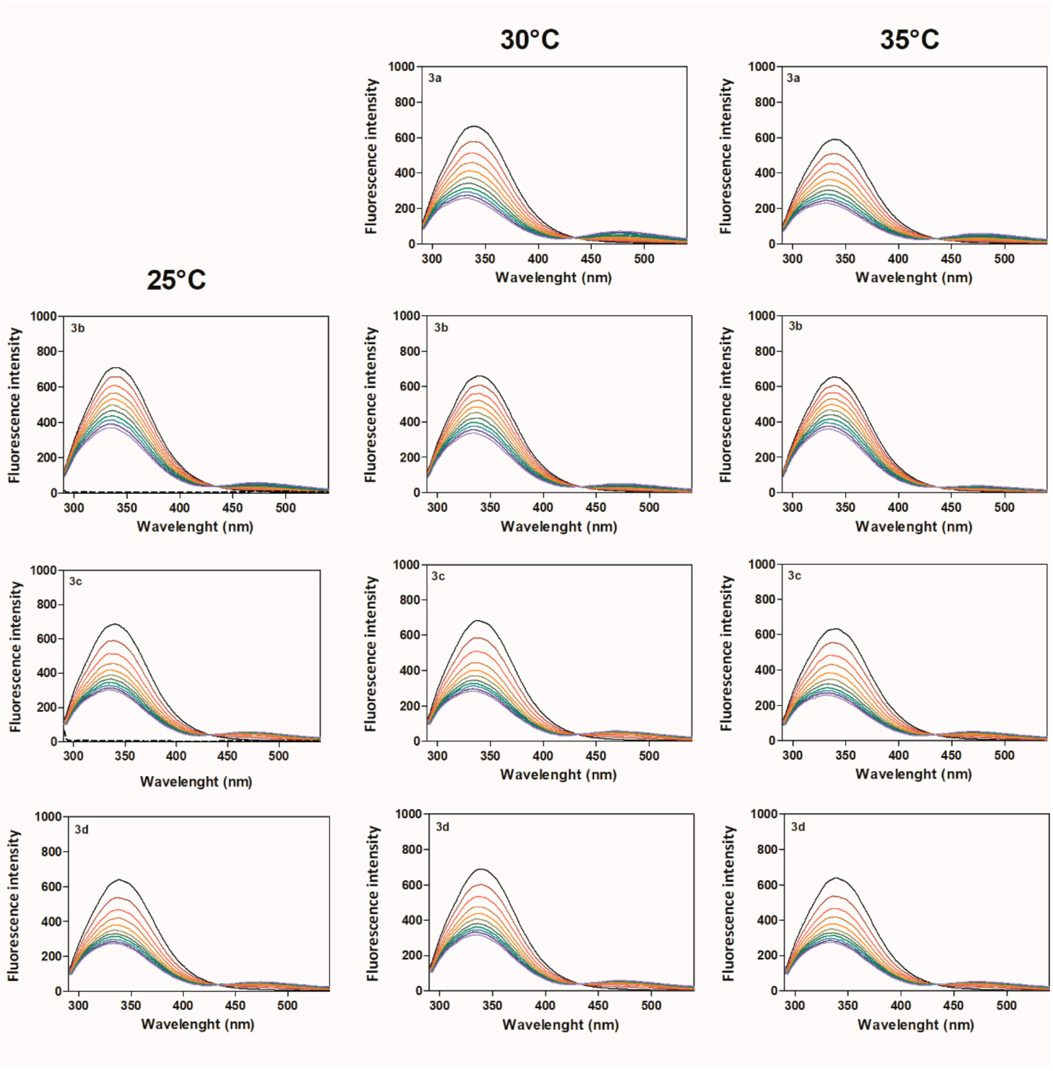

Figure S1. Fluorescence quenching spectra of HSA by different concentration of compound **3a–3d** with λex = 280 nm in Tris-HCl/NaCl buffer, pH = 7.4, at three temperatures 25, 30 and 37 °C. (The plain black line [HSA] = 4 µM; plain color line [**3a–3d**] = 0–6.2 µM).

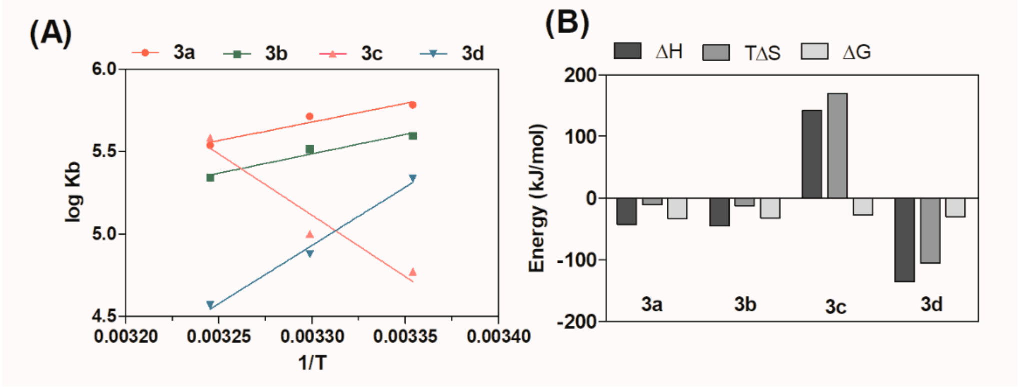

Figure S2. Van´t Hoff plot for the interaction of **3a–3d** (A) and thermodynamic profil (B) with HSA at three temperatures, [HSA] = 4 µM; [**3a–3d**] = 0–6,2 µM.

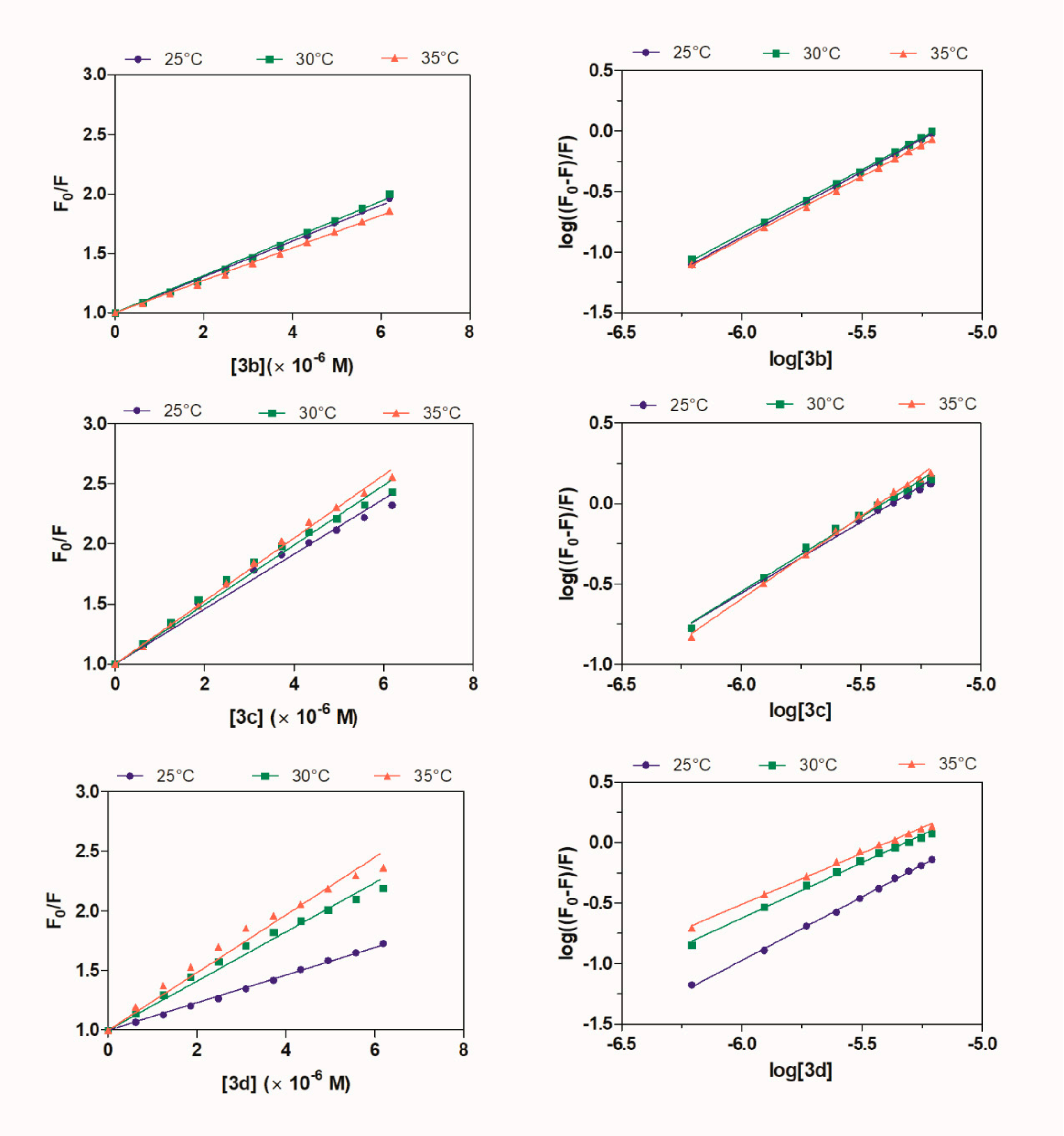

Figure S3. Stern-Volmer plots of HSA + **3b–3d** and double logarithm plot of HSA + **3b–3d** at three differnt temperature. ([HSA] = 4 μM; [**3a–3d**] = 0–6.2 μM).

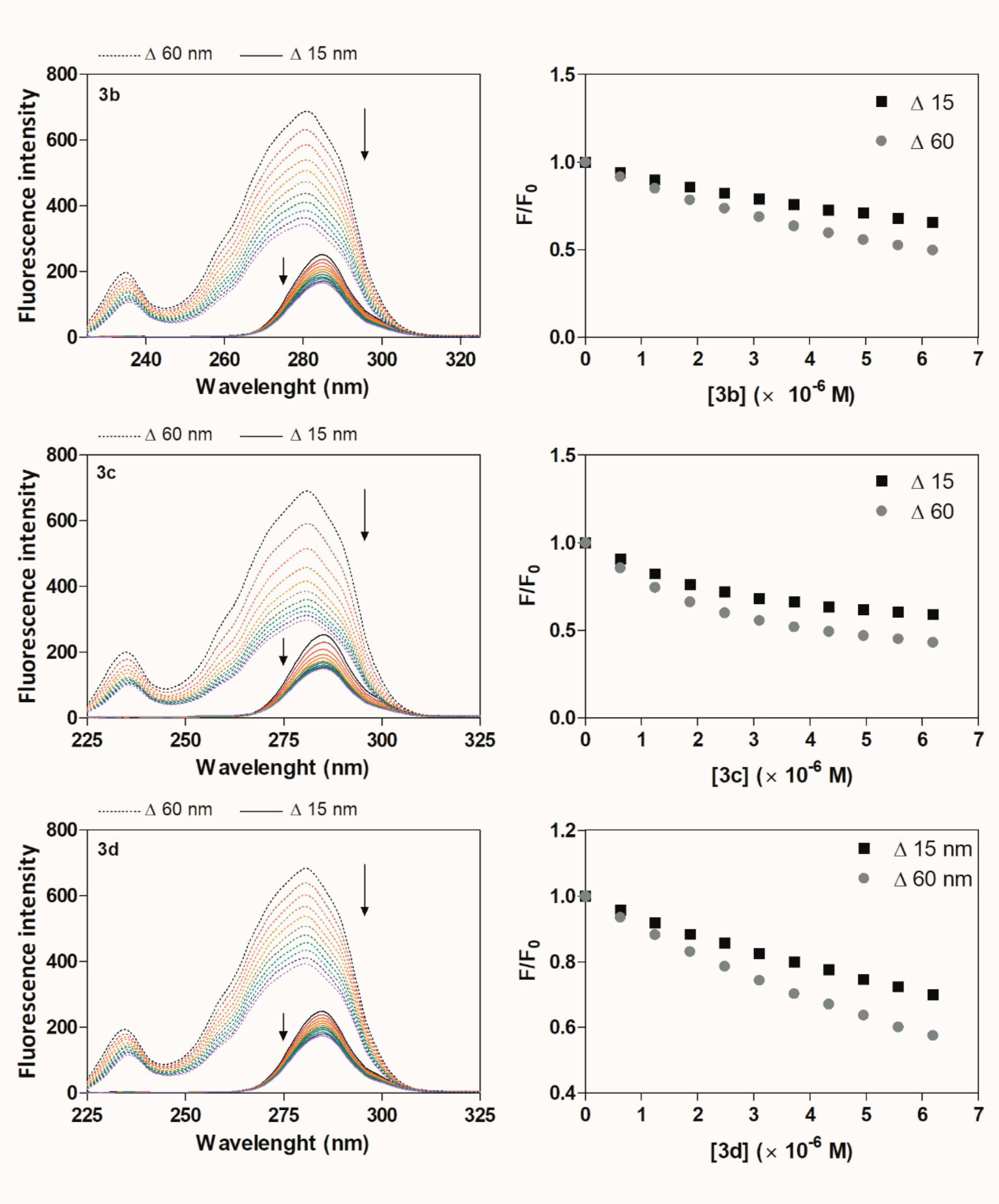

Figure S4. Synchronous fluorescence spectra of HSA + **3b–3d** and corresponding F/F_0 plots at room temperature. ([HSA] = 4 μM; [**3b–3d**] = 0 – 6.2 μM; dashed line for Δλ = 60 nm and plain line for Δλ = 15 nm). Arrow shows the intensity change upon increasing concentration of the quencher.

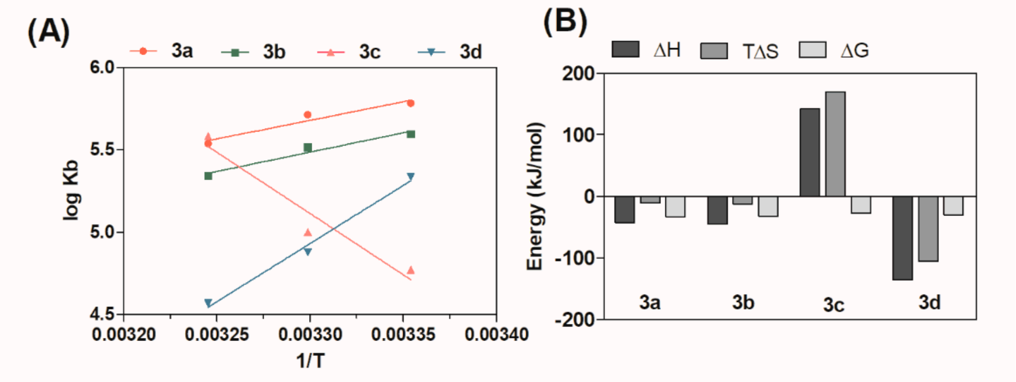

Figure S5. Van´t Hoff plot for the interaction of **3a–3d** (A) and thermodynamic profil (B) with HSA at three temperatures. ([HSA] = 4 μM; [3a-3d] = 0–6,2 μM).

Complex HSA:**3b**

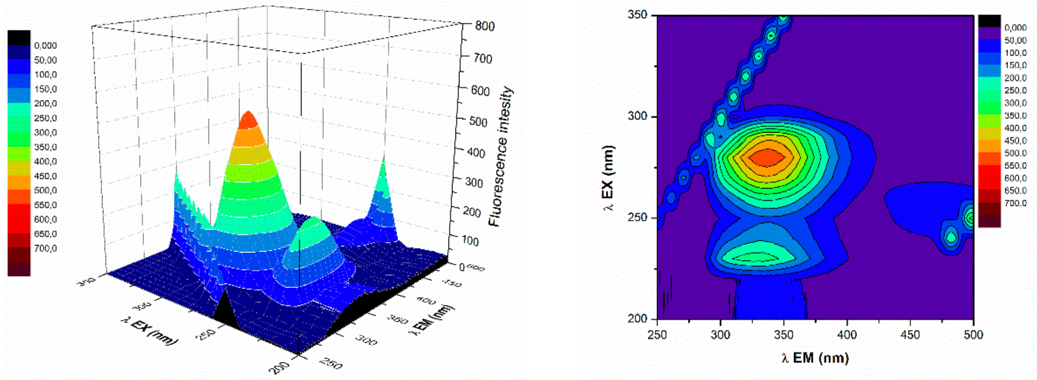

Complex HSA:**3c**

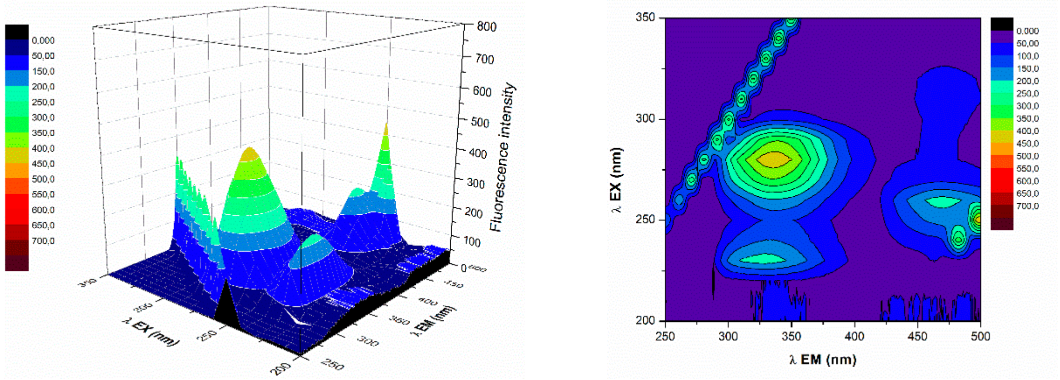

Complex HSA:3d

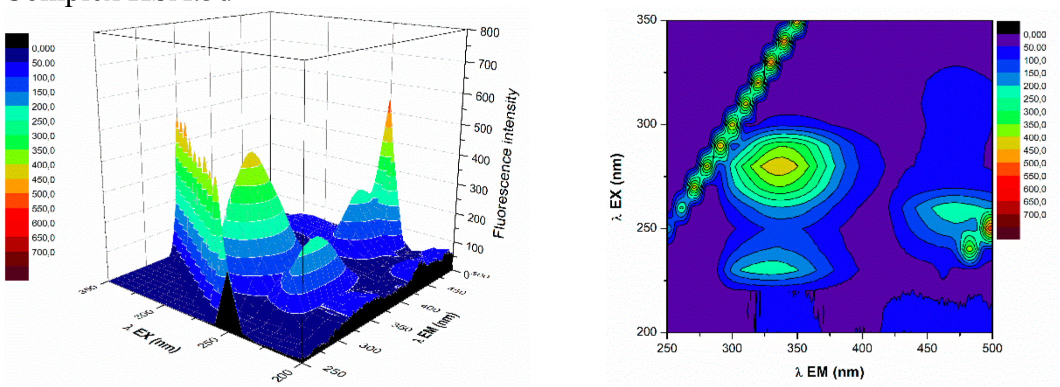

Figure S6. Three-dimensional fluorescence spectra and corresponding contour diagrams of free HSA, HSA:**3b**, HSA:**3c**, HSA:**3d** systems in Tris-HCl/NaCl, pH = 7.4 at 25°C. ([HSA], [**3b**–**3d**] = 4 μM).

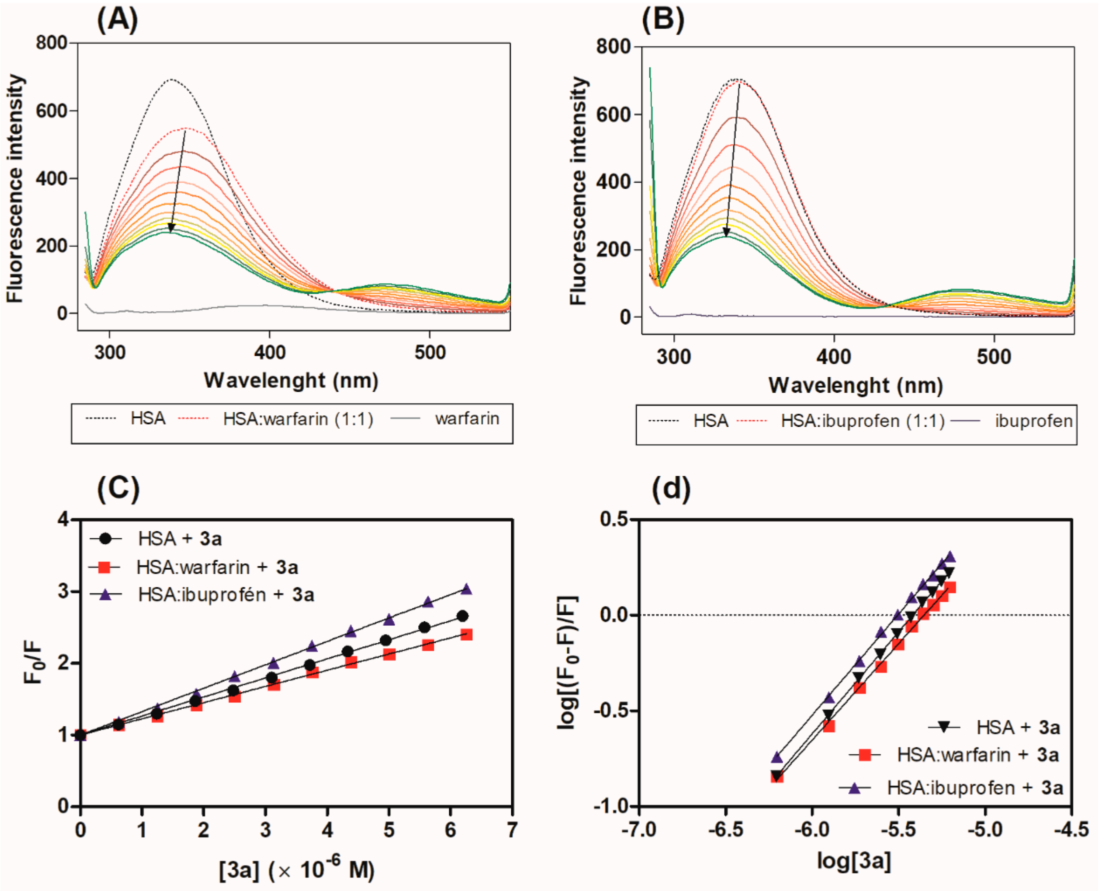

Figure S7. HSA competitive experiments - reverse titration. Emission spectrum (A) HSA:warfarin (1:1), (B) HSA:ibuprofen in presence increasing concentration of **3a** (0.6–6 µM), (C) Stern-Volmer plots and (D) double logarithm plot. ([HSA] = 4 µM).

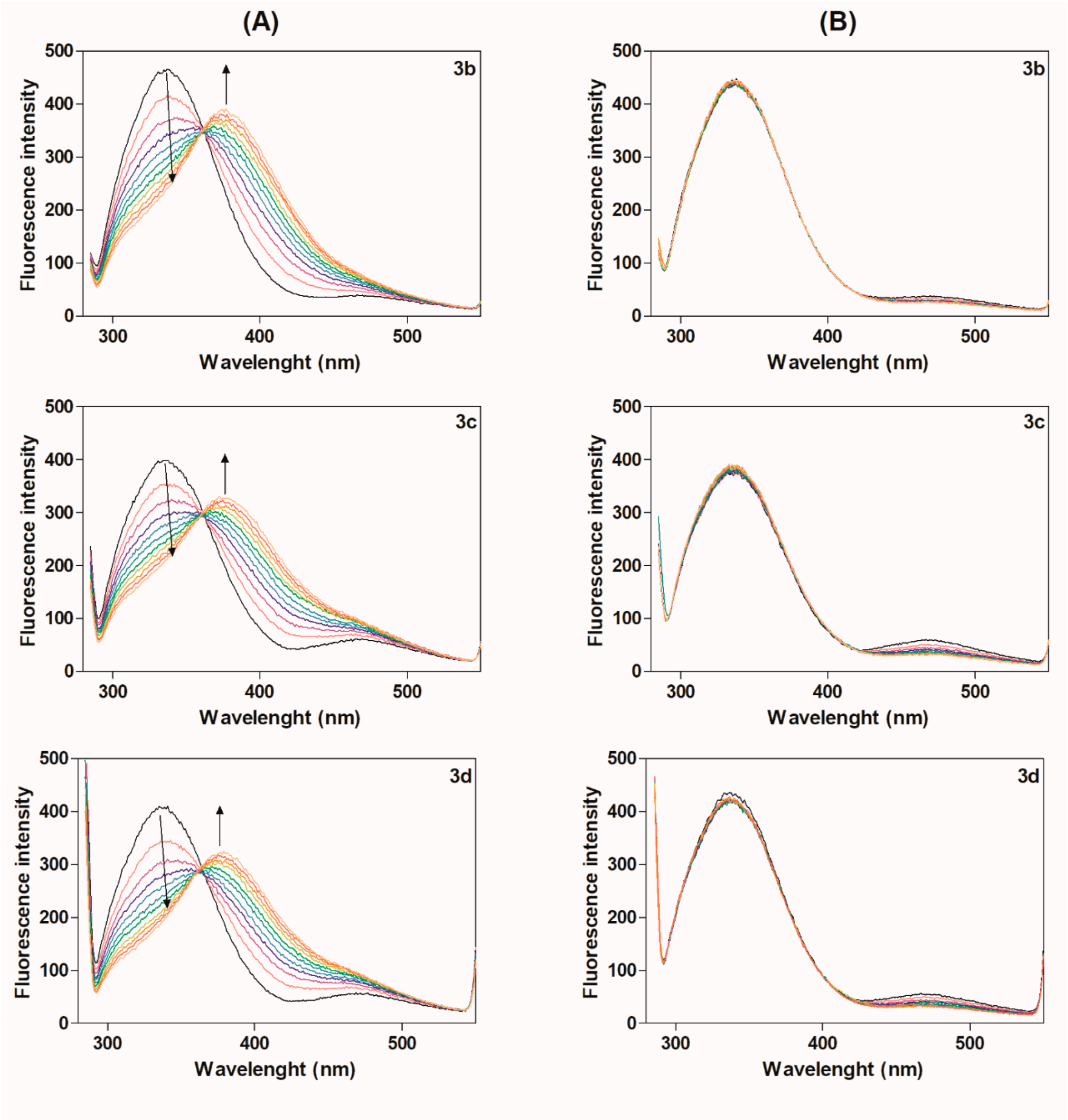

Figure S8. Emission spectrum HSA:acridine derivatives **3b–3d** (1:1, black line) in presence increasing concentration site marker (A) warfarin and (B) ibuprofen (colored line). Molar ratio HAS:marker is: 1:0.5, 1:1, 1:1.5, 1:2, 1:2.5, 1:3, 1:3.5, 1:4, 1:4.5 and 1:5, [warfarin/ibuprofen] = 0–20 µM.
